# Single-Cell Transcriptome Profiles Reveal Fibrocytes as Potential Targets of Cell Therapies for Abdominal Aortic Aneurysm

**DOI:** 10.3389/fcvm.2021.753711

**Published:** 2021-11-24

**Authors:** Bolun Li, Xiaomin Song, Wenjun Guo, Yangfeng Hou, Huiyuan Hu, Weipeng Ge, Tianfei Fan, Zhifa Han, Zhiwei Li, Peiran Yang, Ran Gao, Hongmei Zhao, Jing Wang

**Affiliations:** ^1^State Key Laboratory of Medical Molecular Biology, Department of Pathophysiology, Institute of Basic Medical Sciences, Chinese Academy of Medical Sciences, Peking Union Medical College, Beijing, China; ^2^First Clinical College, Xi'an Jiaotong University, ShaanXi, China; ^3^Department of Basic Medical Sciences, School of Medicine, Tsinghua University, Beijing, China; ^4^State Key Laboratory of Medical Molecular Biology, Department of Physiology, Institute of Basic Medical Sciences, Chinese Academy of Medical Sciences, Peking Union Medical College, Beijing, China

**Keywords:** abdominal aortic aneurysm, single cell sequencing, fibrocytes, cell therapy, cell atlas

## Abstract

Abdominal aortic aneurysm (AAA) is potentially life-threatening in aging population due to the risk of aortic rupture and a lack of optimal treatment. The roles of different vascular and immune cells in AAA formation and pathogenesis remain to be future characterized. Single-cell RNA sequencing was performed on an angiotensin (Ang) II-induced mouse model of AAA. Macrophages, B cells, T cells, fibroblasts, smooth muscle cells and endothelial cells were identified through bioinformatic analyses. The discovery of multiple subtypes of macrophages, such as the re-polarization of *Trem2*^+^*Acp5*^+^ osteoclast-like and M2-like macrophages toward the M1 type macrophages, indicates the heterogenous nature of macrophages during AAA development. More interestingly, we defined CD45^+^COL1^+^ fibrocytes, which was further validated by flow cytometry and immunostaining in mouse and human AAA tissues. We then reconstituted these fibrocytes into mice with Ang II-induced AAA and found the recruitment of these fibrocytes in mouse AAA. More importantly, the fibrocyte treatment exhibited a protective effect against AAA development, perhaps through modulating extracellular matrix production and thus enhancing aortic stability. Our study reveals the heterogeneity of macrophages and the involvement of a novel cell type, fibrocyte, in AAA. Fibrocyte may represent a potential cell therapy target for AAA.

## Introduction

Abdominal aortic aneurysm (AAA) is an aging-related vascular disease. During the progression of AAA, the aneurysm slowly expands and acutely causes vessel rupture without any symptom and warning in advance, resulting in high mortality. For patients with ruptured AAAs, endovascular repair or open repair are the main clinical remedies. However, even undergoing intervention for repair, the in-hospital mortality is still as high as 53.1% (1). New therapies, such cell or stem cell therapies, have been proposed but have encountered difficulties in their development. Therefore, it is necessary to investigate the mechanism of AAA formation and identify the novel biomarkers or therapies to diagnose and treat AAA patients.

Previous studies revealed that AAA was caused by chronic inflammation and an imbalance between synthesis and degradation of extracellular matrix (ECM) composed of elastin and collagen (2, 3). Chronic inflammation is characterized by infiltration of a variety of immune cells. Macrophages have been most widely studied (3, 4) as main inflammatory cells. Besides macrophages, the roles of B cells (5), T cells (6), and mast cells (7, 8) in AAA formation have also been reported, while the function of other immune cells like natural killer (NK) T cells in AAA formation is still rarely studied.

In addition to inflammation, another essential pathological feature of AAA is the dysregulation of ECM proteins that are mainly synthesized by vascular smooth muscle cells (SMCs) and adventitial fibroblasts. SMC apoptosis has been identified as a hallmark of AAA pathology (9). Matrix metalloproteinases (MMPs) are mainly produced by SMCs, fibroblasts and infiltrated macrophages. Increased MMPs lead to matrix degradation, loss of aortic wall integrity, AAA expansion and rupture. The balance of ECM synthesis and degradation is critical for the stability of AAA (2, 10). Fibroblasts, as the major matrix-producing cell population, can transform into myofibroblasts to produce ECMs (11).

In addition to the classical vascular and immune cells, recent evidence suggests that circulating fibrocytes are the bone-marrow derived precursors of fibroblasts and can regulate the ECM (12). Fibrocytes are found in the plaques of atherosclerosis (13). In ischemic cardiomyopathy, fibrocytes can be recruited and involved in the fibrosis (14). Given these possible functions of fibrocytes, they may constitute another essential cell population in AAA formation.

To determine the specific cell types and further explore the molecular mechanisms, we performed single-cell RNA sequencing (scRNA-seq) of mouse AAA tissues, in which different cell types and subtypes in AAA can be defined by unique transcriptomes.

## Materials and Methods

### Animal Model

To induce AAA, 8-week-old *ApoE*^−/−^ male mice (C57BL/6J, Beijing Vital River Laboratory Animal Technology Co., Ltd. Beijing) were anesthetized with 200 mg/kg ketamine and 10 mg/kg xylazine by intraperitoneal injection, and infused with 1,000 ng/kg/min Ang II (A9525-50MG sigma-Aldrich) or saline with alzet osmotic mini-pumps (Alzet model 2004, DURECT Corp., Cupertino, CA) for 28 days. To evaluate the effect of fibrocytes, 8 × 10^6^ fibrocytes were injected into mice via tail vein on days 7 and 21 after Ang II-infusion (1.6 × 10^7^ in total). GFP-labeled fibrocytes were injected with the same protocol to trace them *in vivo*. All animal experiments were approved by the Research Ethics Committee of Peking Union Medical College.

### Single-Cell RNA-seq and Analysis

A single-cell cDNA library was generated as previously described (15). For the isolation of individual cells, both control and AAA mice were anesthetized with ketamine (200 mg/kg) and xylazine (10 mg/kg) by intraperitoneal injection. The normal aorta (one whole aorta from one mouse) or aneurysm tissue (one piece of the supra-renal part with aneurysm from one AAA mouse) were dissected and digested for 15 min at 37°C in PBS containing 200 U/ml collagenase I (SCR103, Sigma Aldrich, Germany), 0.05 U/ml elastase (E1250, Sigma Aldrich, Germany), 5 U/ml neutral protease (LS02111, Worthington, USA), and 0.3 U/ml deoxyribonuclease I (M6101, Promega, USA). The tissue digestion was stopped with DMEM containing 10% FBS (10099141, Gibco, USA.) and the mixture was filtered through a 40 μm cell strainer (15-1040, Biologix, China) to obtain single-cell suspensions. Using the method of 7-amino-actinomycin D (7-AAD) positive cells staining, living aortic cells (7-AAD negative cells) were sorted using a Moflo-XDP (Beckman, USA). Cell suspensions (~10,000 single cells) were next loaded on the Chromium Single Cell Controller (10X Genomics) to generate a single cell and gel bead emulsion (GEM). Single-cell sequencing library preparation was performed according to the instructions of Chromium single cell 3' library & Gel bead kit v2 (10X Genomics). Libraries were sequenced by Illumina Hiseq X Ten in paired-end to reach ~50,000 reads per single-cell (Novo Generation Bioinformatics Technology Co., Ltd.).

The 10X Genomics single-cell transcriptome sequencing data were filtered by removing bases with a mass less than 3 at the beginning and end of the reads, using a CellRanger software suite version 3.0.2 pipeline (16). The filtered reads were aligned to the MM10 mouse reference genome by STAR. For further analysis and statistics, based on the barcode and gene expression matrix, single-cell data were log-normalized and filtered by the R 3.6.0 package Seurat 3.2.3 (17, 18), with the following parameters: unique gene count per cell >500, cell counts per gene >3 (0.1% of the total cell amount), and percentage of mitochondrial genes <0.05. After the control and AAA samples were combined, the UMAP and automated cluster detection algorithms were performed stepwise. The resolution for cluster identification was set as 0.5. DEGs were identified by the Wilcox test with default parameters and *p* <0.05. DEGs from each cluster were sorted by log_e_-fold change relative to the other clusters. Then, the DEGs of which log-fold change was more than 0 were selected and used for GO analysis by ClusterProfiler 3.14.3 (https://guangchuangyu.github.io/software/clusterProfiler/). The enriched function or pathway were further clustered to predict the most likely function of the specific cluster. Alternatively, to detect the possible polarization or differentiation process among different clusters, Monocle 2.14.0 (19) was used for the trajectory analysis. The co-expression level of *n* genes was calculated by $\sqrt[{n}]{e_{1}\times e_{2}\times \cdots \times e_{n}}$ (*e*_*n*_ means the expression of the *n*th gene) and the accuracy is confirmed by ROC curve. Velocyto.R v0.6 package were then used for calculating RNA velocity of each single cells and further visualization.

### Flow Cytometry

For flow cytometry analyses, single-cell suspensions of mouse aortae were prepared according to the previous protocol (20) and stained with following antibodies: 7AAD (00-6993-50, eBioscience, USA), Fluorescent isothiocyanate (FITC) anti-mouse-α-SMA (ab8211, Abcam, United Kingdom), PE-anti-mouse-CD34 (551387, BD, USA), FITC-anti-mouse-CD68 (MA1-82739, Invitrogen, USA), PE-anti-mouse-NK1.1 (ab269324, Abcam, United Kingdom), FITC-anti-mouse-CD19 (11-0193-82, eBioscience, USA), PE-anti-mouse-CD21 (12-0211-82, eBioscience, USA), PE-anti-mouse-CD45 (12-0451-82, eBioscience, USA), and Collagen I (ab88147, Abcam, United Kingdom; at room temperature for 30 min) with Alexa Fluor 488 goat anti-mouse antibody (A32723, Invitrogen, at room temperature for 30 min). Flow cytometry results were analyzed by using FlowJo 7.6 software (FlowJo, LLC., Ashland, Oregon). Statistical analysis was further performed by GraphPad Prism 8.0.

### Isolation and Culture of Bone Marrow-Derived Fibrocytes

Bone marrow-derived fibrocytes were obtained by *in vitro* differentiation of spleen cells as previously described (21). Spleen cells were isolated from 8-week *ApoE*^−/^^−^ or generic GFP male mice as previously described (22). Briefly, spleens were separated, minced and digested at 37°C. Spleen cells were isolated using a 70 μm cell strainer (15-1070, Biologix, China) and cultured in 3 ml of RPMI-1640 (31800-500, Solarbio, China) with L-Glutamine, 20% fetal bovine serum (10099-141, Gibco, Australia), penicillin–streptomycin (P1400, Solarbio, China). After 3 days, the medium was changed into Fibrolife basal media (LM-0001, Lifeline Cell Technology, USA) with 25 ng/ml of murine macrophage colony-stimulating factor (M-CSF) (315-02, PeproTech, USA) and 50 ng/ml of murine interleukin (IL)-13 (210-13, PeproTech, USA) and cells were cultured in a humidified incubator containing 5% CO_2_ at 37°C for 10 days. To detect whether cells were induced differentiation, we observed the morphological changes under microscopy (Nikon, Tokyo, Japan). Following 10 to 14 days, the adherent cells were harvested and used to injected to mice and immunofluorescence staining.

### Human Aortic Tissue

Human aortic tissue extracts were prepared from the abdominal aortic aneurysm patient (Peking Union Medical College Hospital and Beijing Anzhen Hospital) and from the body of the deceased donor with no detectable vascular disease (Peking Union Medical College Volunteer Corpse Donation Reception Station). Age and gender were matched (Supplementary Tables S1, S2). The human donor aortic tissues obtained were approved by the institutional review board of institute of basic medical sciences, Chinese academy of medical sciences. The abdominal aortic aneurysm tissue obtained were approved by the institutional review board of Peking Union Medical College Hospital and Beijing Anzhen Hospital.

### Hematoxylin and Eosin (H&E) Staining and Elastin Staining

The mouse aortae were embedded in optical cutting temperature (OCT) compound (4583, Sakura, Netherlands) and the frozen sections were used for H&E staining, Elastin staining or immunofluorescence staining. H&E staining and Elastin staining were performed by using H&E staining kit (G1120, Solarbio, China) elastic staining Kit (HT25A, Sigma-aldrich, Germany) according to the manufactures' instructions.

### Immunofluorescence Staining

For immunofluorescence staining, the sections for mouse aortae and human aortae or cells seed in 35 mm glass bottom dish were fixed with 4% paraformaldehyde for 10 min and permeabilized with 1% Triton X-100 for 15 min. Then the sections blocked with 1% bovine serum albumin for 30 min at room temperature and incubated with mouse monoclonal collagen I (ab88147, Abcam, United Kingdom, 1:100 dilution) and rabbit polyclonal CD45 (ab10558, Abcam, United Kingdom, 1:100 dilution) antibodies overnight at 4°C. After washing with PBS three times, sections were incubated Alexa Fluor 488-conjugated goat anti-mouse IgG (H+L) secondary antibody (A-11008, Invitrogen, USA, 1:1,000), Alexa Fluor 594 conjugated goat anti-rabbit IgG(H+L) highly cross-adsorbed secondary antibody (A-11037, Invitrogen, USA, 1:1,000 dilution) for 40 min at room temperature. Finally, sections were mounted with ProLong Gold antifade reagent with DAPI (ab104139, Abcam, United Kingdom). Pictures were taken by confocal microscopy (LSM780, Zeiss).

### Quantification and Statistical Analysis

For animal experiments, the exact number of mice used in each experiment is reported in the figure legends. Normality tests were conducted first. Then, comparisons between two groups were made with an unpaired two-tailed *t*-test or non-parametric test. Comparisons among groups with two factors were made with two-way ANOVA, and *p* < 0.05 was considered statistically significant. Data were presented as mean ± standard error of the mean (SEM).

## Results

### Aortic and Aneurysmal Cell Populations Revealed by scRNA-seq

**Figure 1:**
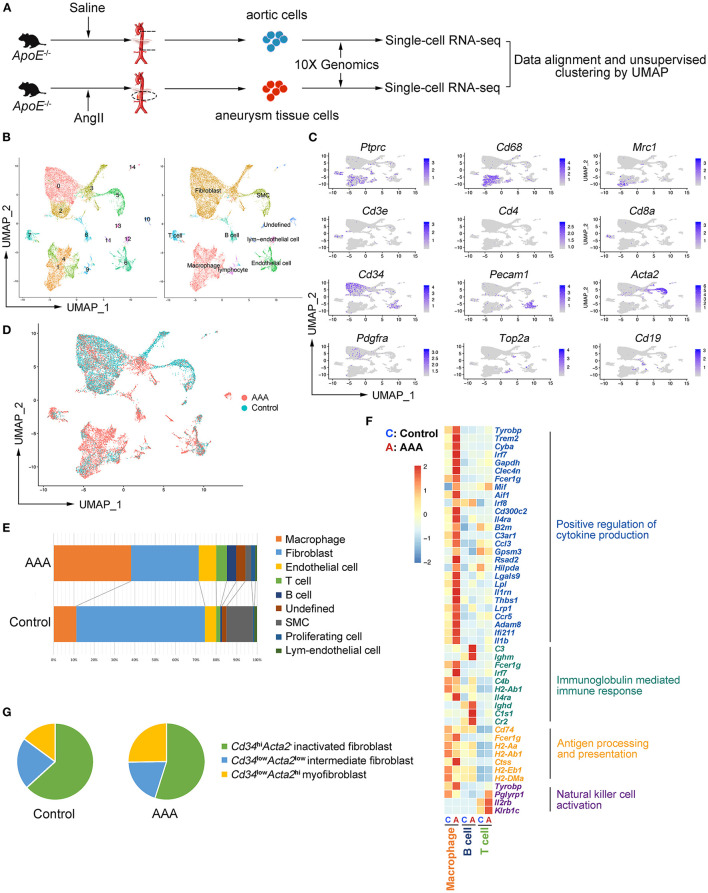
Aortic and aneurysmal cell populations revealed by scRNA-seq. **(A)** Schematic diagram of the experimental design. **(B)** Uniform manifold approximation and projection (UMAP) representation of the aligned gene expression data from saline-treated (control, *n* = 7,914, in blue) and Ang II-treated (AAA, *n* = 9,338, in red) aortae of apolipoprotein E (*ApoE*^−/−^) mice, showing partition of 15 distinct clusters (left panel) and cell identities (right panel). **(C)** The expression of marker genes exhibited on UMAP plot (gene expression log-normalized by Seurat). **(D)** UMAP representation of cells showing cellular origins. **(E)** Stacked column chart of the proportion of each identified cell type in control and AAA group individually (Control vs. AAA). **(F)** Heatmap of key genes differentially expressed after Ang II infusion (in the left panel) and the associated Gene Ontology (GO) pathway enrichment (in the right panel). Heatmap showed the relative expression level in each cluster. **(G)** Pie chart of the proportion of fibroblast subtypes in control and AAA groups (Control vs. AAA). SMC: smooth muscle cell.

To explore the cell types involved in AAA, apolipoprotein E–deficient (*ApoE*^−/−^) male mice were implanted with Ang II osmotic pump for 4 weeks to induce AAA or underwent sham surgery, and the tissues were collected for scRNA-seq (Figure 1A). After alignment (Supplementary Figure S1A) and quality control (Supplementary Figures S1B,C), 7,914 and 9,338 cells from control and AAA groups were combined and included in the subsequent analysis. Unsupervised clustering by Seurat R package revealed 15 distinct clusters (Figure 1B). In accordance with the canonical markers, the clusters were fibroblasts (Cluster 0, 2, 3; *Cd34*^+^*Pdgfra*^+^), macrophages [Cluster 1, 4; *Cd68*^+^*Ptprc*^+^ (encoding *Cd45*)], SMC [Cluster 5; *Acta2*^+^ (encoding α-SMA) *Cd34*^−^*Pdgfra*^−^], endothelial cells (ECs) [Cluster 6; *Pecam1*^+^ (encoding *Cd31*), T cells (Cluster 7; *Cd3e*^+^*Cd8a*^+^), B cells (Cluster 8; *Cd19*^+^), lymphatic endothelial cells (Cluster 12; *Lyve1*^+^*Pecam1*^+^), and proliferating cells (Cluster 9; *Top2a*^+^*Mki67*^+^)] (Figure 1C). Consistent with previous studies, among all cell types, the proportion of immune cells significantly increased in the AAA group, especially macrophages and B cells (Figures 1D,E). By contrast, fibroblasts and SMCs were reduced after 4-week of Ang II-exposure (Figures 1D,E).

To assess the robustness of the dataset and explore the cellular mechanisms of AAA formation, differentially expressed gene (DEG) were determined by comparing each cell type between AAA and control groups and Gene Ontology (GO) analyses were performed. We found that the functions of “positive regulation of cytokine production,” “regulation of inflammatory response,” and “antigen processing and presentation” (Figure 1F; Supplementary Figure S2A) was upregulated in macrophages in AAA. Moreover, B cells in this dataset were predicted to be activated to plasma cells with the high expression level of *Cr2* (encoding *Cd21*) and *Ighm*, and the upregulated genes of B cells were mainly enriched in the function of “immunoglobulin mediated immune response.” As for T cells, “natural killer cell activation” was upregulated with increased expression of *Klrb1c* and *Il2rb* in AAA pathogenesis. To validate the results from scRNA-seq analyses, the cells were analyzed by flow cytometry, which confirmed the infiltration of macrophages, and the activation of B cells and NK T cells in AAA (Supplementary Figures S3A–C).

Fibroblasts were composed of three clusters (Clusters 0, 2 and 3). The highly expressed genes (HEGs) among these clusters and potential functions of these cells were analyzed. The typical fibroblast functions such as “extracellular matrix organization” and “extracellular structure organization” were enriched in all of the three clusters. HEGs of Cluster 0 were mainly enriched in the function of “transmembrane receptor protein serine/threonine kinase signaling pathway” (Supplementary Figure S2B). By contrast, the specifically enriched GO functions of Clusters 2 and 3 were “connective tissue development” and “wound healing” (Supplementary Figures S2C,D). Further analysis based on the gene expression patterns determined Cluster 0 as the inactivated fibroblasts, with higher expression of *Cd34* but no expression of *Acta2* (Supplementary Figure S2E). By contrast, Cluster 3, with higher expression level of *Acta2* and lower expression level of *Cd34* compared with Cluster 0, was regarded as myofibroblasts (11). Cluster 2 could be the intermediate types between Cluster 0 and 3. In addition, scRNA-seq data showed that the proportion of *Acta2*^+^ myofibroblasts increased significantly (Figure 1G) in AAA, which was validated by flow cytometry analysis (Supplementary Figure S3D).

In terms of vascular cells, SMCs involved in the function of “regulation of apoptotic signaling pathway” were upregulated in AAA, which was consistent with the increased SMC death in AAA (Supplementary Figure S2E). Furthermore, ECM-related functions were enriched in SMCs in the AAA group, suggesting that the SMCs underwent phenotypic transformation in AAA (Supplementary Figure S2E). In addition, GO analysis of DEGs of ECs suggested that the upregulated functions were related to leukocyte migration and EC proliferation (Supplementary Figure S2F), which was consistent with the previous reported function of ECs in AAA formation.

### Heterogeneity and Re-polarization of Macrophage Subtypes in AAA

**Figure 2:**
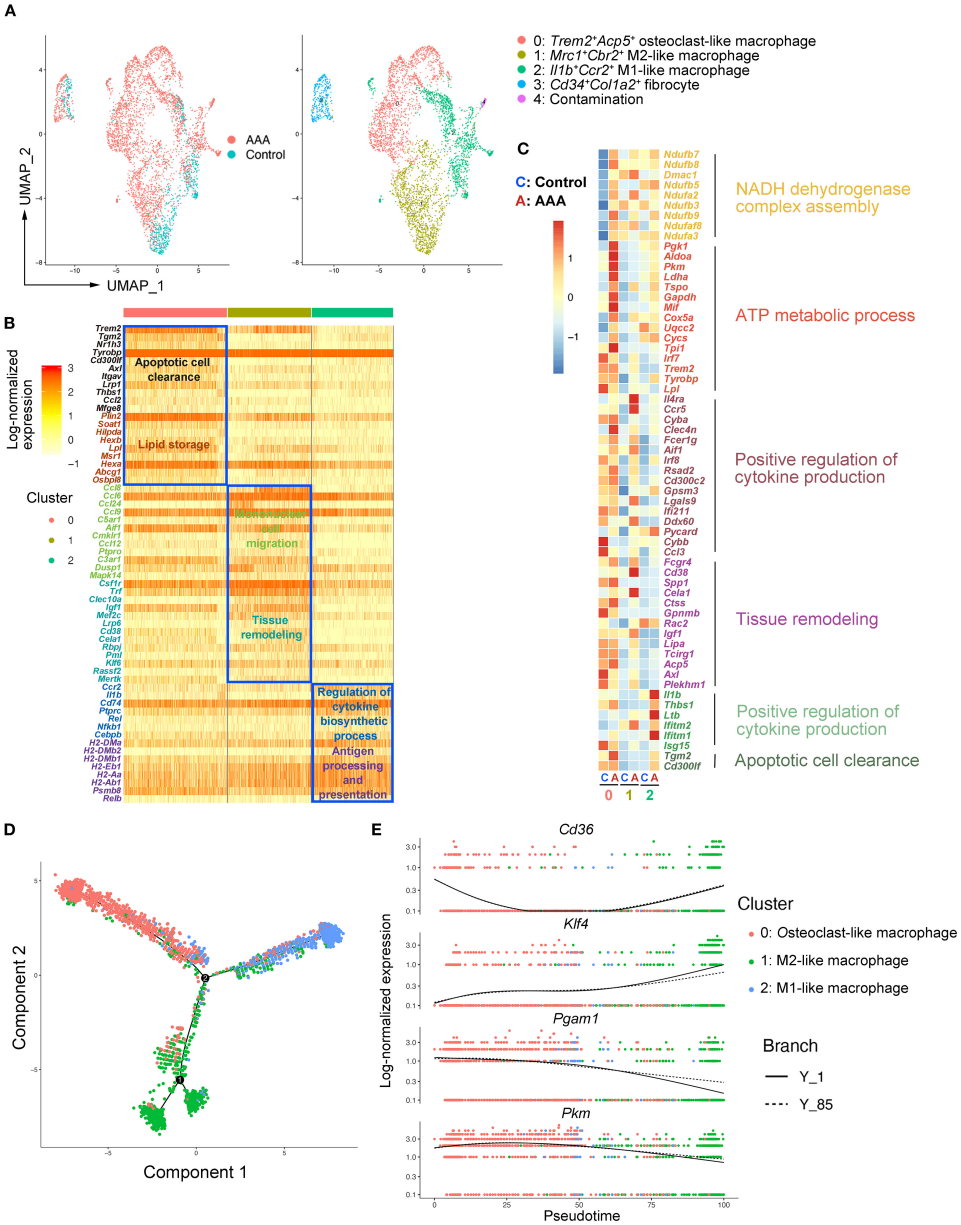
Heterogeneity and re-polarization of macrophage subtypes in AAA. **(A)** Uniform manifold approximation and projection (UMAP) representation of the aligned gene expression data in macrophages separated from Figure 1B. **(B)** Heatmap of highly expressed genes (HEGs) from three subtypes of macrophages in **(A)**. **(C)** Heatmap of key genes differentially expressed (in the left panel) after Ang II infusion and the associated GO pathway enrichment (in the right panel). Heatmap showed the relative expression level in each cluster of macrophages. **(D)** Pseudo-time plot by trajectory analysis of three clusters of macrophages (Cluster 0: *Trem2*^+^*Acp5*^+^ macrophages; Cluster 1: *Mrc1*^+^*Cbr2*^+^ M2-like macrophages; Cluster 2: *Il1b*^+^*Ccr2*^+^ M1-like macrophages). **(E)** The expression of key genes in pseudo-time plot.

As the largest and most varied population in our scRNA-seq data, we focused on macrophages (Clusters 1 and 4) and re-clustered them by Seurat (Figures 1C–E). We observed four main clusters (Clusters 0–3) (Figure 2A). Using canonical markers and HEGs to analyze the macrophage subtypes, we identified *Trem2*^+^*Acp5*^+^ osteoclast-like macrophages (Cluster 0), *Mrc1*^+^*Cd163*^+^ M2-like macrophages (Cluster 1), *Il1b*^+^*Ccr2*^+^ M1-like macrophages (Cluster 2), and *Cd34*^+^*Col1a2*^+^ bone marrow-derived fibrocyte (21, 23) (Cluster 3) (Figure 2A; Supplementary Figure S4A). To define the specific gene expression features and potential functions, HEG and GO analyses were performed on three subtypes of macrophages (Figure 2B). Osteoclast-like macrophages (Cluster 0) expressing high levels of *Trem2, Tyrobp, Plin2*, and *Lpl* were enriched in the function of “apoptotic cell clearance” and “lipid storage,” suggesting that osteoclast-like macrophages could be involved in lipid deposition and the phagocytosis of apoptotic cells or lipid. In M2-like macrophages (Cluster 1), C-C motif chemokine (CCL) genes (*Ccl8, Ccl6, Ccl24, Ccl9* and *Ccl12*) were highly expressed, and their enriched function was “mononuclear cell migration,” implying that M2-like macrophages might contribute to the recruitment of mononuclear cells by producing chemokines in AAA pathogenesis. Furthermore, the enriched function of “tissue remodeling” related genes were also highly expressed in M2-like macrophages, which is consistent with the previous reported phenotypic characteristics of M2 macrophages (24). In M1-like macrophages (Cluster 2), HEGs were mainly enriched in the function of “regulation of cytokine biosynthetic process” and “antigen processing and presentation.” Combining with the high expression of *Il1b* and *Ccr2*, these cells have been predicted to be the most important pro-inflammatory cells in AAA pathogenesis.

To further analyze the changes of macrophage subtypes between control and AAA groups, DEG and GO analyses were performed on each macrophage subtype. In osteoclast-like macrophages, the genes upregulated in AAA were enriched in the function of “NADH dehydrogenase complex assembly,” “ATP metabolic process,” and “glycolytic process” (Figure 2C; Supplementary Figure S4B). In M2-like macrophages, the function of “positive regulation of cytokine production” and “tissue remodeling” were upregulated (Figure 2C). On the other hand, upregulated pro-inflammatory factors, such as *Il1b* and *Thbs1*, were enriched in the function of “positive regulation of cytokine production” in M1-like macrophages. In addition, the expression of genes related to “apoptotic cell clearance” were also increased in M1-like macrophages. More interestingly, we found that the mode of ATP metabolism in osteoclast-like macrophages might be shifted to glycolysis (Supplementary Figure S4B), which is typical of M1 macrophages (25). Moreover, the upregulated functions of M2-like macrophages were similar to the signature functions of M1-like macrophages. These data together suggest a connection between the three subtypes, which was clarified by trajectory analysis with the Monocle2 R package. As shown in Figure 2D, we found the tendency of transition among different macrophages. As shown by the RNA velocity, we found that osteoclast-like macrophages and M2-like macrophages tended to polarize to M1-like (Supplementary Figures S5A,B). Key genes related to the macrophage polarization were significantly changed among these three subtypes of macrophages. For example, *Klf4* (26) was highly expressed in M2 compared to M1 cells and was reported to play a crucial role in macrophage polarization (Figure 2E). Deficiency of *Klf4* could evoke a partial loss of M2 but gain of M1. Moreover, the expression of *Cd36* declined from M2 to M1 (Figure 2E), which was proven to be essential for fatty acid uptake and metabolism of M2 macrophages (27). By contrast, the expression of *Pkm* and *Pgam1* related to glycolysis and gluconeogenesis, increased during the M2 to M1 transition (Figure 2E). The high levels of *Pkm* and *Pgam1* in M1 macrophages indicate that M1 macrophages are glycolytic (25), which was consistent with our GO analysis above (Figure 2C). Taken together, we identified three subtypes of macrophages in aortic and aneurysmal tissues and we thus proposed that the osteoclast-like and M2-like macrophages might polarize toward M1 type in AAA pathogenesis.

### Identification of Fibrocytes That Were Distinct From Macrophages in AAA

**Figure 3:**
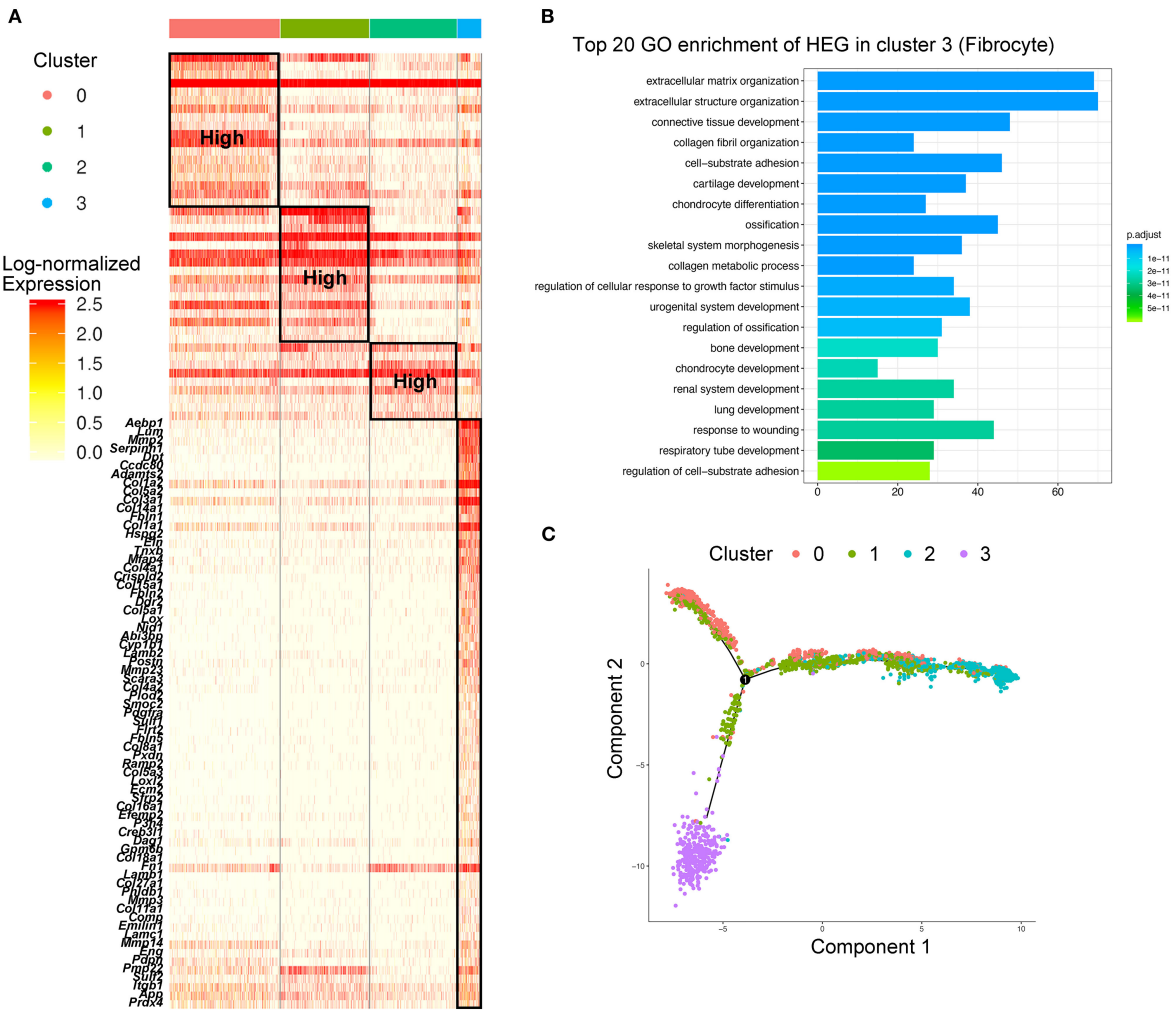
Identification of fibrocytes that were distinct from macrophages in AAA. **(A)** Heatmap of highly expressed genes (HEGs) from three subtypes of macrophages and fibrocytes (Cluster 3), The HEGs of fibrocytes compared with macrophage subtypes were labeled (lower part), and the HEGs of macrophage subtypes (upper part) were the same as Figure 2B. **(B)** Bar plot of Gene Ontology (GO) pathway enrichment of the HEGs in fibrocytes. **(C)** Pseudo-time plot by trajectory analysis of three clusters of macrophages (Cluster 0: *Trem2*^+^*Acp5*^+^ macrophages; Cluster 1: *Mrc1*^+^*Cbr2*^+^ M2-like macrophages; Cluster 2: *Il1b*^+^*Ccr2*^+^ M1-like macrophages; Cluster 3: *Cd34*^+^*Col1a2*^+^ fibrocytes).

While analyzing the subtypes of macrophages, a remarkable population of *Cd34*^+^*Col1a2*^+^ bone marrow-derived fibrocytes (Cluster 3) were found in control aorta and AAA tissues, and tended to increase in AAA (Figure 2A; Supplementary Figures S6A,B). When performing HEG analysis on fibrocytes compared with the three subtypes of macrophages, we found that the expression pattern of fibrocytes was exceedingly distinct from macrophages (Figure 3A). ECM-related genes were highly expressed in fibrocytes, such as collagen (*Col1a1, Col1a2, Col3a1, Col5a, Col4a1*, etc.), elastin (*Eln*), matrix metalloproteinases (*Mmp2, Mmp3*, etc.), and periostin (*Postn*) (Figure 3A). GO analysis of HEGs suggested fibrocyte functions such as ECM organization, collagen related process and response to growth factor and wounding (Figure 3B), similar to the functions of fibroblasts (Supplementary Figures S2B–D). Further trajectory analysis of fibrocytes and macrophages revealed that there might be no connection or transition between the fibrocytes and three subtypes of macrophages (Figure 3C). Taken together, we identified fibrocytes in AAA tissues, whose gene expression patterns and functions were different from those of macrophages.

### Identification and Function of Fibrocytes in AAA Pathogenesis

**Figure 4:**
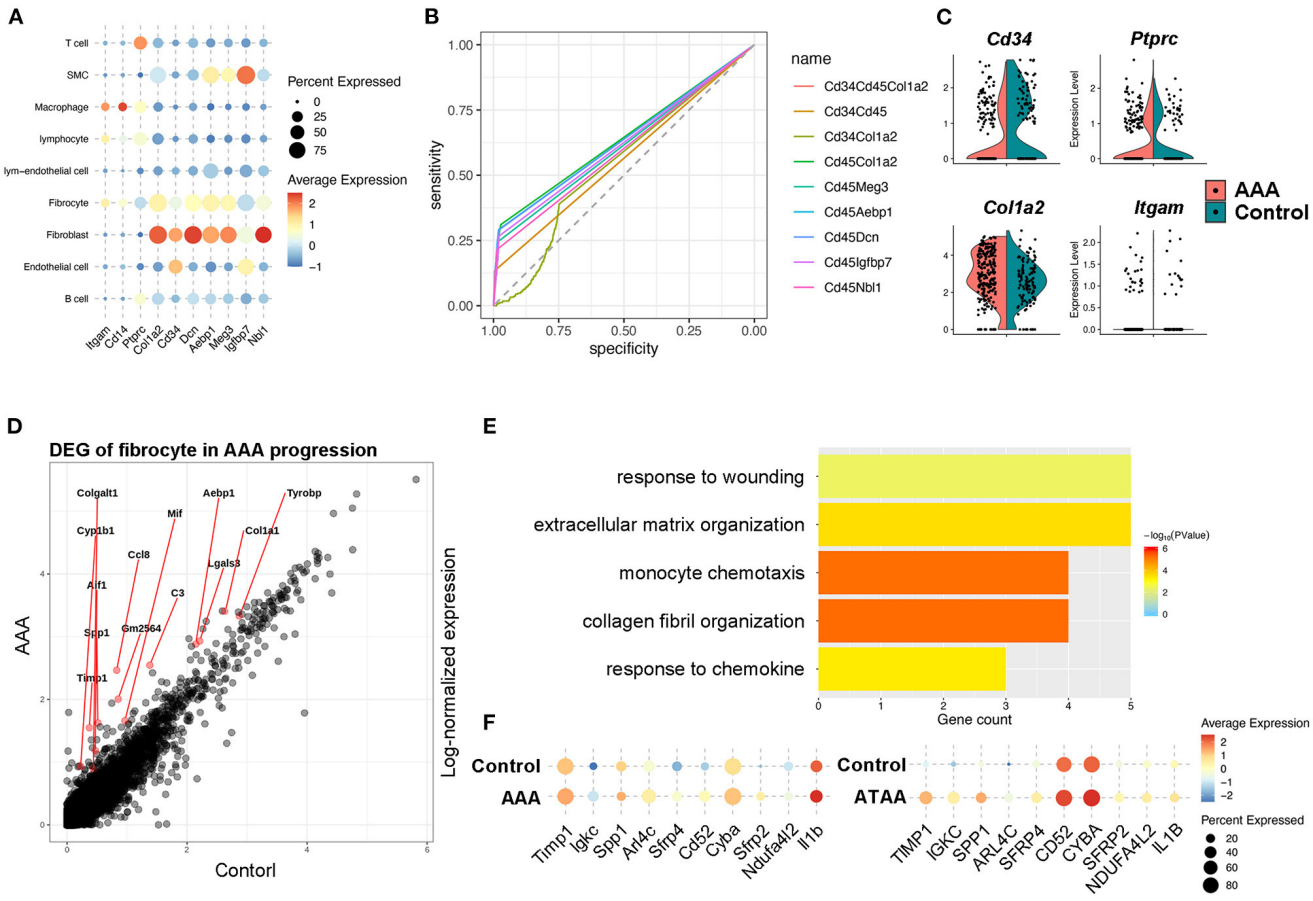
Identification and function of fibrocytes in AAA pathogenesis. **(A)** Dot plot showing the expression of classic fibrocyte markers and the top 5 highly expressed genes compared with macrophages. **(B)** The ROC curve identifying the sensitivity and specificity of the co-expression of distinct marker genes. **(C)** Violin plots of the expression levels of the classic marker genes that identify fibrocytes during Ang II infusion. **(D)** Differentially expressed genes (DEGs) of fibrocytes during Ang II infusion. **(E)** Bar plot of Gene Ontology (GO) pathway enrichment of DEGs in fibrocytes. **(F)** Dot plot showing the expression of 10 shared DEGs in Ang II model and human ATAA.

Bone marrow–derived fibrocytes are differentiated from circulating CD14^+^ peripheral blood mononuclear cells (PBMCs) and co-express markers of hematopoietic stem cells (CD34^+^), monocyte lineage (CD11b^+^), leukocyte (CD45^+^), and stromal cells (collagen I^+^). These characterizations are used to identify fibrocytes (23, 28). In our scRNA-seq analysis, marker genes distinguishing fibrocytes from macrophages were retrieved and combined with the canonical markers to identify fibrocytes from other cell types. Among all cell types, the fibrocyte features distinct from macrophages were remarkably similar with fibroblasts, but the fibrocytes also exhibited leukocyte-like features. This suggests that fibroblasts represent a unique cell type (Figure 4A). For screening the markers of fibrocytes, we calculated the co-expression levels (details in Methods) of the fibroblast features (i.e., *Cd34, Col1a2, Meg3, Aebp1, Dcn, Igfbp7*, and *Nbl1*) and the leukocyte-like features (*Cd45*) and assessed the accuracy and specificity of different feature combinations by ROC curve. The result showed that the combination of *Cd45* and *Col1a2* showed a high specificity (>95%) for identifying fibrocytes with a co-expression level of more than 0.25 (Figure 4B). Fibrocytes were newly identified by co-expression of *Ptprc* and *Col1a2*, and distributed among macrophage, fibroblast and smooth muscle cell clusters (Supplementary Figures S6C,D). Furthermore, we adopted and analyzed a previous single cell RNA-seq dataset from human ascending thoracic aortic aneurysm (29) (ATAA) to validate our current findings in mice. By calculating the co-expression level of *PTPRC* and *COL1A2*, fibrocytes were also detected in the ATAA dataset. Fibrocytes were mainly distributed among macrophage, fibroblast and smooth muscle cell clusters in the embedding space (Supplementary Figures S7A,B), which is consistent with our results. In addition, the proportions of fibrocytes tended to increase in ATAA patients (Supplementary Figure S7C). Next, the transcriptomic changes of fibrocytes in AAA were analyzed. In terms of the classic markers of fibrocytes, the hematopoietic stem cell- and immune cell-like features (i.e., *Cd34, Ptprc, Itgam*) were decreased but the stromal feature (*Col1a2*) was increased (Figure 4C). DEG analysis of our data showed the upregulation of *Ccl8, Gm2564, Aif1*, and *Lgals3* in fibrocytes, which were enriched in the function of “monocyte chemotaxis.” The expression levels of *Timp1, Tyrobp, Mif, Col1a1, and C3* were increased, and they were enriched in the function of “response to wounding” (Figures 4D,E). These data together suggest the response and recruitment of fibrocytes to the AAA lesion. In addition, ECM and collagen-related functions were upregulated in fibrocytes during AAA formation (Figure 4E), which were similar to fibroblast functions (Supplementary Figures S2B–D). Further combining with the ATAA single cell dataset, DEGs of fibrocytes caused by aortic aneurysms were overlapped with those in our dataset and determined. Specifically, *Timp1, Sfrp2*, and *Sfrp4* were upregulated in both human and mouse samples (Figure 4F). These genes are known to be closely related to AAA formation (30–32). Further trajectory analysis between fibroblasts and fibrocytes showed fibrocytes were similar with inactivated fibroblasts and might be differentiated toward myofibroblasts (Supplementary Figure S5D), consistent with their role as the fibroblast progenitor. Taken together, we hypothesized that fibrocytes are recruited to AAA tissues and tend to transform toward fibroblasts to influence AAA pathogenesis by mediating ECMs remodeling.

**Figure 5:**
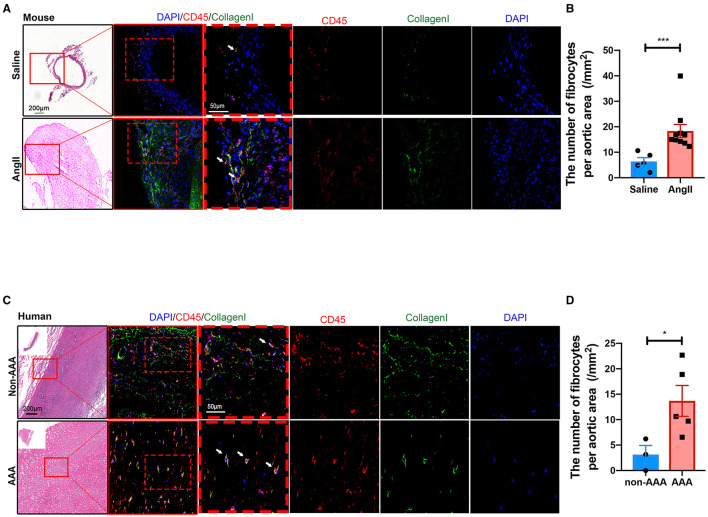
Validation and localization of fibrocytes in AAA. **(A)** Representative images of H&E staining and the immunofluorescence images of normal aorta and AAA tissue from mice stained with COL1 (green) and CD45 (red). **(B)** Quantification of the number of fibrocytes per aortic area (/mm^2^). The quantitative data were shown as mean ± SEM, and the difference between the groups was evaluated by Mann-Whitney test. (Saline: *n* = 5; Ang II: *n* = 10). **(C)** Representative images of H&E staining and the immunofluorescence images of normal aorta and AAA tissue from human stained with COL1 (green) and CD45 (red). **(D)** Quantification of the number of fibrocytes per aortic area (/mm^2^). The quantitative data were shown as mean ± SEM, and the difference between the groups was evaluated by Mann-Whitney test. *p < 0.05, and ***p < 0.001. (non-AAA: *n* = 3; AAA: *n* = 5).

In order to validate the existence and localization of fibrocytes, co-immunofluorescence staining was performed and the results showed that numerous CD45^+^COL1^+^ fibrocytes were significantly increased in both mouse (Figures 5A,B) and human (Figures 5C,D) AAA tissues, suggestive of fibrocytes mainly in the adventitia. Co-localization of CD45 and elastin layers showed that CD45^+^ cells were mainly localized in the adventitia (Supplementary Figure S8), further indicating the localization of fibrocytes in the adventitia. Flow cytometry data further confirmed the increase of fibrocytes in mouse AAA tissues (Supplementary Figure S9).

### Alleviation of Ang II-Induced AAA Formation by Reconstitution of Fibrocytes

**Figure 6:**
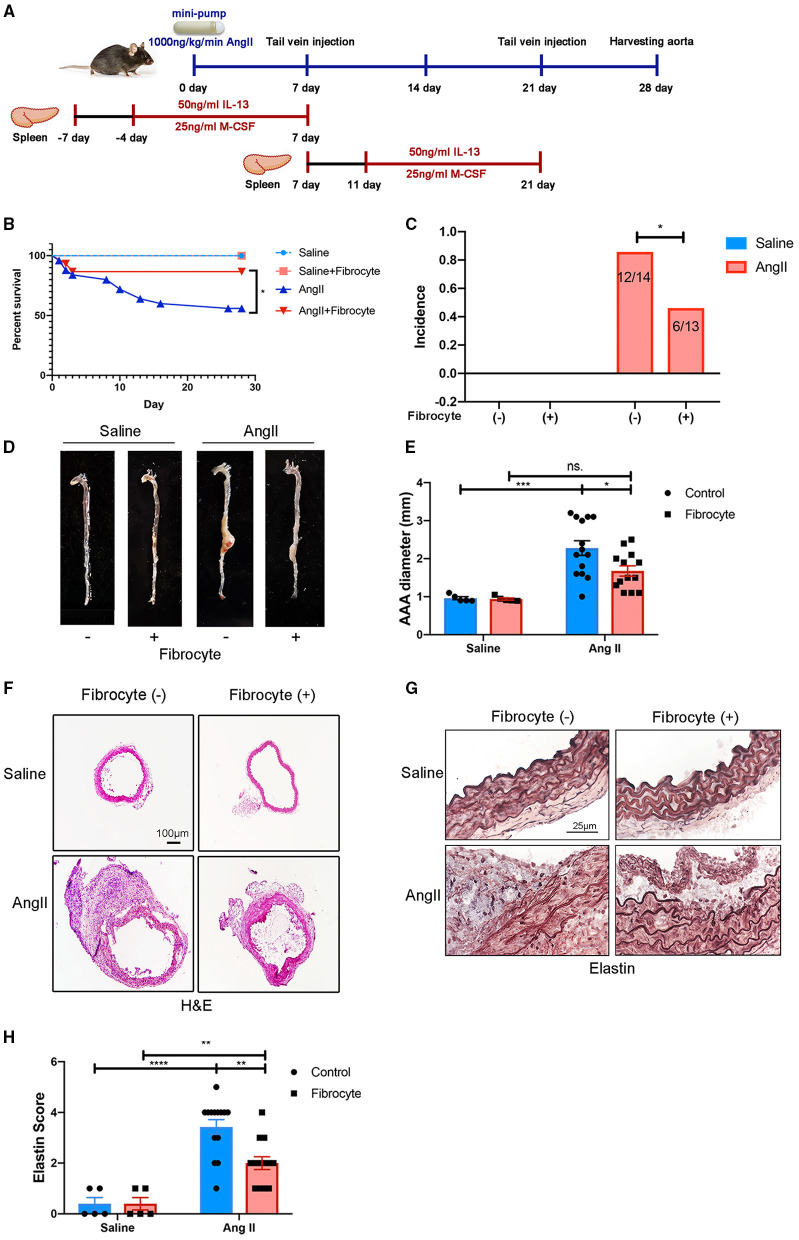
Alleviation of Ang II-induced AAA formation by reconstitution of fibrocytes. **(A)** Schematic diagram of the fibrocytes reconstitution experiment. **(B)** Survival curves of control and Ang II-infused mice with or without fibrocytes reconstitution [Fibrocyte (–): *n* = 5 for control mice and *n* = 25 for Ang II-infusion mice; Fibrocyte (+): *n* = 5 for control mice and *n* = 15 for Ang II-infusion mice]. **(C)** The incidence rate of AAA in control and Ang II-infused mice with or without fibrocytes reconstitution. Fisher exact method was used to test for statistical significance. **(D)** Representative images of whole aortae. **(E)** Quantification of AAA diameter (mm). **(F)** Representative images of H&E staining of abdominal aortae. Scale bar = 250 μm. **(G)** Representative images of elastin staining in four groups of mice. Scale bar = 25μm. **(H)** Quantification of the elastin score. The quantitative data were shown as mean ± SEM, and the difference between multiple groups was evaluated by two-way ANOVA. *p < 0.05, **p < 0.01, ***p < 0.001, and ****p < 0.0001. [Fibrocyte (–): *n* = 5 for control mice and *n* = 14 for Ang II-infusion mice; Fibrocyte (+): *n* = 5 for control mice and *n* = 13 for Ang II-infusion mice].

Fibrocytes have been reported to play essential roles in wound healing, atherogenesis, and lung diseases (21, 23, 33, 34), but their role in AAA is not understood. To test the hypothesized roles of fibrocytes in AAA formation, bone marrow-derived fibrocytes were administered into Ang II-treated mice on days 7 and 21 by tail-vein injection (Figure 6A). Bone marrow-derived fibrocytes were produced from spleen monocytes and cultured with IL-13 (50 ng/ml) and M-CSF (25 ng/ml) for 10 days (Supplementary Figure S10). GFP-labeled fibrocytes from GFP-transgenic mice were then injected into Ang II-infused mice (non-GFP) to trace the cells *in vivo*. By co-staining GFP with fibrocyte markers, GFP-labeled fibrocytes were found to be recruited to AAA tissues (Supplementary Figure S11). More importantly, reconstitution of fibrocytes significantly attenuated AAA formation, demonstrated by a decrease in AAA incidence and mortality (Figures 6B,C), reduced lesion diameters (Figures 6D–F) and diminished elastin degradation (Figures 6G,H). Taken together, our data suggest that fibrocytes attenuate Ang II-induced AAA formation.

## Discussion

We performed scRNA-seq analysis of aortic tissues from Ang II-induced AAA mouse model and found infiltration and activation of pro-inflammatory cells such as macrophages, B cells and NK T cells in the AAA tissue. Additionally, myofibroblast activation, SMC apoptosis, and EC dysfunction were also indicated by the results of scRNA-seq. We focused on the heterogeneity of macrophage subtypes, and discovered the repolarization of M2-like and *Trem2*^+^ osteoclast-like macrophages toward M1-like macrophages, highlighting a critical role of this process in AAA pathogenesis. More importantly, we identified fibrocytes from scRNA-seq analysis and confirmed the recruitment of fibrocytes and their protective effect in AAA formation. Thus, our study has identified specific cell populations critical to AAA and provided evidence supporting these cells as targets for intervention.

Despite the rapidly growing number of single-cell sequencing studies, only a few single-cell datasets of AAA have been published, including data from ATAA patients (29), the elastase model (35) and the CaCl_2_ model (36). Hadi et al. conducted scRNA-seq on Ang II-induced AAA tissues and showed the expression of Nertin-1 in different cell types without in-depth analysis on the heterogeneity of cell populations. We performed scRNA-seq in Ang II-induced AAA tissues and obtained consistent findings with previously reported results. For example, B cells have been reported to play a crucial role by producing abundant inflammatory factors in AAA (37). Depletion of B cells using anti-CD20 could suppress the formation of AAA and evoke the infiltration of immunosuppressive cells (38). Consistently, we found the activation and increase of B cells, which was confirmed by flow cytometry in AAA. Similarly, the activation of NK T cells has been demonstrated to aggravate AAA formation (39), which is also consistent with our scRNA-seq and flow cytometry results. Apoptosis of SMCs has become the hallmark of AAA, which is also confirmed in our scRNA-seq data in terms of cell proportions and function analysis. Moreover, both previous studies and our scRNA-seq results showed the vital function of ECs in the recruitment of inflammatory cells during AAA formation. The consistent results from our and previous studies support the important roles of these cell types in AAA.

Our scRNA-seq analysis revealed the heterogeneities of three macrophage subtypes and found the increase of *Trem2*^+^ osteoclast-like macrophages in AAA tissues. These data were consistent to the previous reports that osteoclast-like macrophages were increased in AAA lesions, and the inhibition of osteoclastogenic differentiation diminished AAA formation (40–42). In addition, based on the gene expression patterns and trajectory analysis, we proposed the re-polarization of osteoclast-like macrophages and M2-like macrophages toward the M1 type. However, it remains unclear when and how re-polarization takes place in AAA formation, which deserves to be further investigated. Besides promoting inflammation (Supplementary Figure S2A), infiltrated macrophages can also influence ECM degradation by secreting proteases, such as cathepsins and MMPs (43, 44). It has been reported that cathepsin S was highly expressed in AAA lesions, and deficiency of cathepsin S attenuated AAA formation by preventing SMC apoptosis and proliferation of inflammatory cells. Consistently, our scRNA-seq data showed an upregulated expression of cathepsins in different macrophages. Specifically, cathepsins S and B were most highly expressed in osteoclast-like macrophages and the expression levels were increased in AAA (Supplementary Figure S6A). Similarly, the expression of a series of cathepsins was upregulated in M2-like macrophages, including *Ctsa, Ctsb, Ctsc, Ctsd* (Supplementary Figure S12). In addition, previous studies have reported that MMP9 and MMP12 produced by macrophages contribute to AAA (45, 46). Our data showed that *Mmp9* was highly expressed in M2-like macrophages, and *Mmp12* and *Mmp14* were highly expressed in osteoclast-like macrophages correspondingly (Supplementary Figure S6A). These data together suggest the vital roles of different types of macrophages in AAA formation by differentially regulating inflammation and ECMs remodeling.

Fibrocytes are bone marrow-derived cells maturing in spleen. Fibrocytes are recruited to injured sites and differentiated toward fibroblasts (23). Previous studies have proposed their roles in wound healing, atherosclerogensis, and lung diseases (21, 33, 34, 47, 48). Reconstitution of fibrocytes could accelerate wound healing by upregulating collagen-related genes (23). Similarly, reconstitution of *Postn*^−/−^ fibrocytes diminished bleomycin-induced lung fibrosis (48). In vascular pathology, fibrocytes were found to be involved in the atherosclerotic plaque formation (33, 47). In our study, the reconstitution of fibrocytes reduced the incidence and mortality of Ang II-induced AAA, and attenuated elastin degradation and AAA formation. In our study, we found the activation of fibroblast toward myofibroblast with high *Acta2* expression in AAA (Figure 1G). Importantly, single cell trajectory analyses revealed fibrocytes, as another source, tended to be differentiated toward fibroblasts and then myofibroblasts (Supplementary Figure S5D). The results suggested that the increased myofibroblasts in the AAA group may result from the activation of adventitial fibroblast as well as the recruited and increased fibrocytes. In previous studies, the activation and differentiation of fibroblast toward myofibroblast is a well-established phenomenon during which fibroblasts mature and acquire the contractile feature. This phenotypic transition contributes to vascular pathology by producing inflammatory factors, such as IL-6 and monocyte chemoattractant protein-1 (49, 50). In terms of the source of myofibroblasts, they have been reported to be differentiated from not only fibroblasts, but also from SMCs and fibrocytes (51), which are consistent with our observations. For the underlying mechanism of transition process, previous studies have revealed the roles of TGFβ, hypoxia, angiotension II, endothelin-1, IL-6, hyperhomocysteinemia (HHcy), cylindromatosis (CYLD), nicotinamide adenine dinucleotide phosphate (NADPH) oxidase 4 (Nox4), and Fizzl (50, 52–55). The enhanced expression of ECM-related genes (i.e., *Col1a1* and *Timp1*) and WNT antagonists (i.e. *Sfrp2* and *Sfrp4*) in fibrocytes (Figure 4F) suggests a protective effect in AAA formation. Additionally, these genes are involved in the TGFβ pathway (56), indicating the transition of fibrocytes to myofibroblasts. The specific mechanisms by which fibrocytes modulate AAA formation need to be further characterized in the future.

In conclusion, this study identified important roles of macrophage subtypes and fibrocytes in AAA by scRNA-seq. Reconstitution experiment with cell tracing confirmed that fibrocytes were recruited and attenuated AAA formation. As clinical treatment for AAA lacks breakthrough, our data provide the theoretical underpinning for fibrocytes as a new approach for cell therapy for AAA treatment or post-operative protection, where fibrocytes can maintain aortic homeostasis and reduce the mortality due to AAA. Additionally, human fibrocytes can be easily derived from PBMCs for autologous cell therapy, providing a new and feasible strategy for future treatment of AAA.

## Limitation

This study has some limitations. First, we have one AAA mouse versus one saline control for scRNA-seq. Multiple biological replicates in scRNA-seq will more conductive to exploring the heterogeneities of cell types in AAA. However, we did validation experiments to confirm cell heterogeneity in our scRNA-seq data, which were consistent with other scRNA-seq studies (29, 35, 36). The changes among the different cell types in AAA were confirmed by flow cytometry (Supplementary Figure S3). The involvement of fibrocytes in AAA pathogenesis were confirmed by reconstitution experiments in mice (Figure 6). Nevertheless, the results suggested that the cell types analyzed by scRNA-seq data were critical in progression of AAA. Second, the cell types captured and their proportions might be affected by the different enzyme digestion strategies used for obtaining single-cell suspensions, as shown in the previous scRNA-seq studies of vessel tissues (29, 35, 36). In our study, based on our preliminary experiments, we optimized the enzymatic strategy [PBS containing 200 U/ml collagenase I (Sigma Aldrich), 0.05 U/ml elastase (Sigma Aldrich), 5 U/ml neutral protease (Worthington), and 0.3 U/ml deoxyribonuclease I (Promega)]. Our results showed that the cell types and the proportions were similar with the single cell dataset of the CaCl_2_ model (36). In addition, the cleanliness of dissected adventitia can also affect the cell types and its proportions. In this study, we kept more adventitial tissue to acquire complete cell atlas of the aorta, which might have resulted in more fibroblasts in this dataset. Taken together, these factors may have caused the relatively low numbers of vascular smooth muscle cells in the control saline group. Therefore, the method of obtaining single-cell suspensions should be continually optimized in future studies. Finally, we only performed scRNA-seq study in Ang II-induced AAA mouse model. Different animal AAA models show multiple differences in anatomic positions, histological features, and pathological mechanisms. None of the experimental rodent models developed to date is exactly identical to human AAAs. Therefore, our findings need to be confirmed in AAAs from different animal models and perhaps human tissues in the future.

## Data Availability Statement

The datasets presented in this study can be found in online repositories. The names of the repository/repositories and accession number(s) can be found at: https://ngdc.cncb.ac.cn/gsa/PRJCA006049.

## Ethics Statement

The studies involving human participants were reviewed and approved by the Institutional Review Board of Peking Union Medical College Hospital and Beijing Anzhen Hospital. The patients/participants provided their written informed consent to participate in this study. The animal study was reviewed and approved by the Research Ethics Committee of Peking Union Medical College.

## Author Contributions

BL, XS, RG, and JW designed the study. XS, RG, HZ, YH, WGu, WGe, HH, and TF performed the experiments. BL, HZ, XS, ZH, and ZL analyzed the data. BL, PY, HZ, and JW wrote the manuscript. All authors read and approved the final manuscript.

## Funding

This study was financially supported by Chinese Academy of Medical Sciences Innovation Fund for Medical Sciences (2021-1-I2M-016) (to RG); the National Natural Science Foundation of China Grants 82100514 (to RG); the National Key Research and Development Program of China grants 2019YFA0801804 (to JW); Thousand Young Talents Program of China (to JW).

## Conflict of Interest

The authors declare that the research was conducted in the absence of any commercial or financial relationships that could be construed as a potential conflict of interest.

## Publisher's Note

All claims expressed in this article are solely those of the authors and do not necessarily represent those of their affiliated organizations, or those of the publisher, the editors and the reviewers. Any product that may be evaluated in this article, or claim that may be made by its manufacturer, is not guaranteed or endorsed by the publisher.
