# Supplementary material for: Single-Cell Transcriptome Profiles Reveal Fibrocytes as Potential Targets of Cell Therapies for Abdominal Aortic Aneurysm
Source: Front Cardiovasc Med. 2021 Nov 24;8:753711. doi: 10.3389/fcvm.2021.753711 (PMC8652037; doi:10.3389/fcvm.2021.753711)
Supplement: Supplementary file 1 [file Data_Sheet_1.docx]

Supplementary Material

**This file includes:**

**Supplementary Tables**

Table S1. Information for human subjects

Table S2. Human subject characteristics

**Supplementary Figures**

Figure S1. Quality control of scRNA-seq data.

Figure S2. GO analysis of DEGs of different cell types in scRNA-seq data.

Figure S3. FACS analysis of cell heterogeneities during AAA pathogenesis.

Figure S4. Identification of macrophage subtypes.

Figure S5. Re-polarization of macrophage subtypes and fibroblasts during AAA pathogenesis.

Figure S6. Newly identified fibrocytes by co-expressing *Ptprc* and *Col1a2* in AAA dataset.

Figure S7. Validation of fibrocytes in human ATAA dataset.

Figure S8. Localization of CD45^+^ cells in aorta.

Figure S9. Flow cytometry analysis of fibrocytes during AAA progression

Figure S10. Culture of bone marrow-derived fibrocytes.

Figure S11. GFP-labeled fibrocyte tracing in reconstitution tissue.

Figure S12. Expression of Cathepsins and matrix metalloproteinases in macrophage subtypes.

**Table S1. Information for human subjects (n = 8)**

| Variable | AAA1 | AAA2 | AAA3 | AAA4 | AAA5 | non-AAA1 | non-AAA2 | non-AAA3 |
| --- | --- | --- | --- | --- | --- | --- | --- | --- |
| Gender | male | male | male | male | male | male | male | male |
| Age(year) | 53 | 43 | 65 | 63 | 47 | 35 | 82 | 85 |
| Diagnosis | AAA | AAA | AAA | AAA | AAA | donor | donor | donor |
| Hypertension | Yes | Yes | Yes | No | No | No | No | No |
| Smoking status | Current | Current | Never | Current | Never | Current | Never | Never |

**Table S2. Human subject characteristics**

|  | non-AAA (3) | AAA (5) | *p*-value |
| --- | --- | --- | --- |
| Male (%) | Yes (100%) | Yes (100%) | *ns* |
| Age$\pm$SEM | 67.3$\pm$16.19 | 5$4.2\pm$4.32 | 0.5714 |
| Hypertension (%) | Yes (0%) | Yes (60%) | *ns* |
| Smoking status (%) | Current (33%) | Current (60%) | *ns* |

Data were shown as mean$\pm$stardard error of mean (SEM) or frequencies.


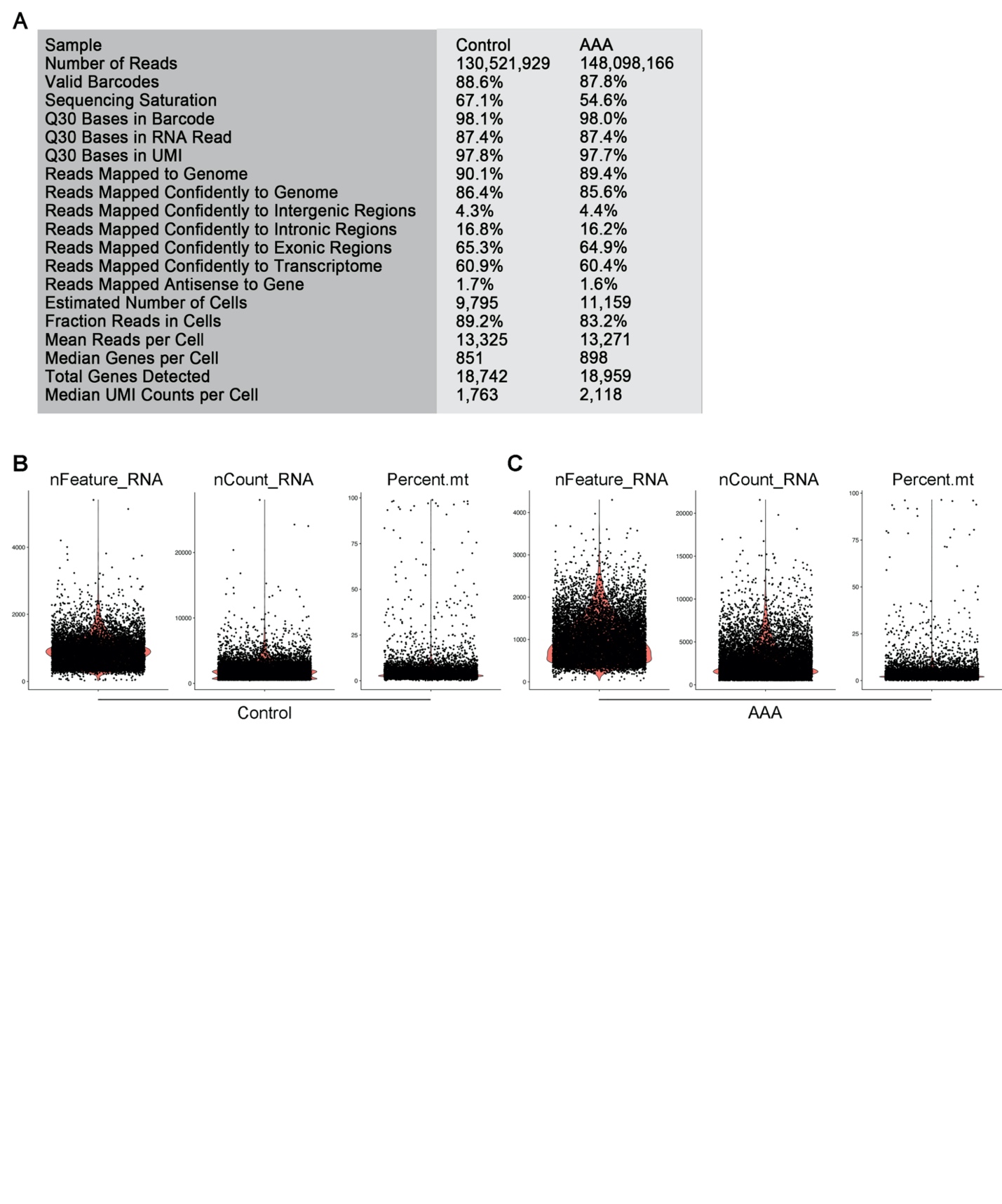


**Supplementary information, Fig. S1. Quality control of scRNA-seq data.**

**(A)** Statements of scRNA-seq alignment and quality control summarized by CellRanger 3.0.2 before filtered by Seurat. **(B-C)** Basic information of scRNA-seq data in control and AAA group (labeled under the graphs) summarized by Seurat (nFeature_RNA: the number of expressed genes in each cell; nCount_RNA: the total expression level of each cell; Percent.mt: percentage of mitochondrial genes occupied total genes).

_
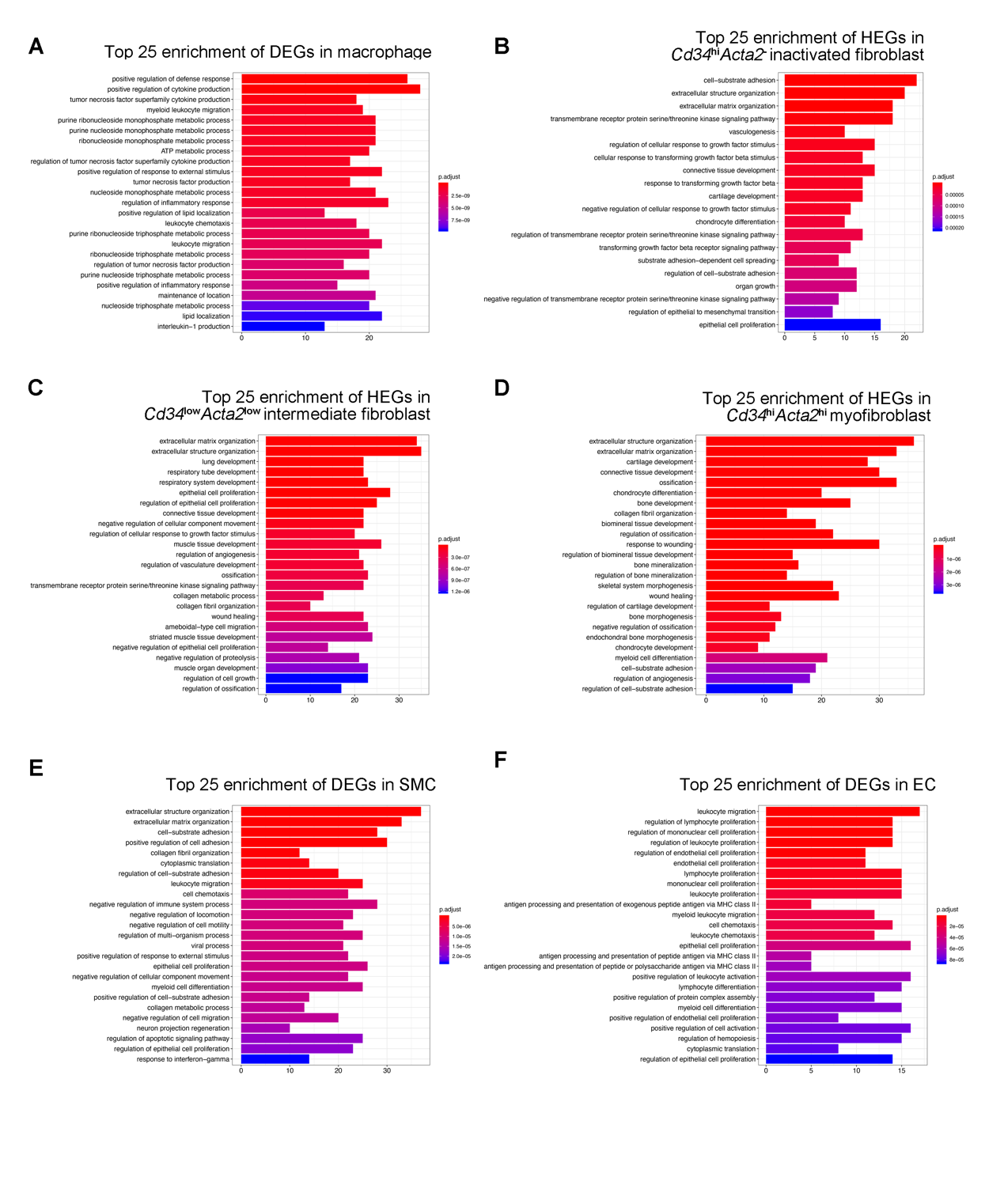
_

**Supplementary information, Fig. S2. GO analysis of HEGs of different cell types in scRNA-seq data.**

**(A)** Gene ontology (GO) pathway enrichment bar plot of differentially expressed gene (DEGs) in macrophages. **(B-D)** GO pathway enrichment bar plot of highly expressed genes (HEGs) in *Cd34^hi^Acta2^-^* inactivated fibroblast (**B**), *Cd34^low^Acta2^low^* intermediate fibroblast (**C**), and *Cd34^hi^Acta2^hi^* myofibroblast (**D**). **(E-F)** GO pathway enrichment bar plot of DEGs in smooth muscle cell (SMC; **E**) and endothelial cell (EC; **F**).


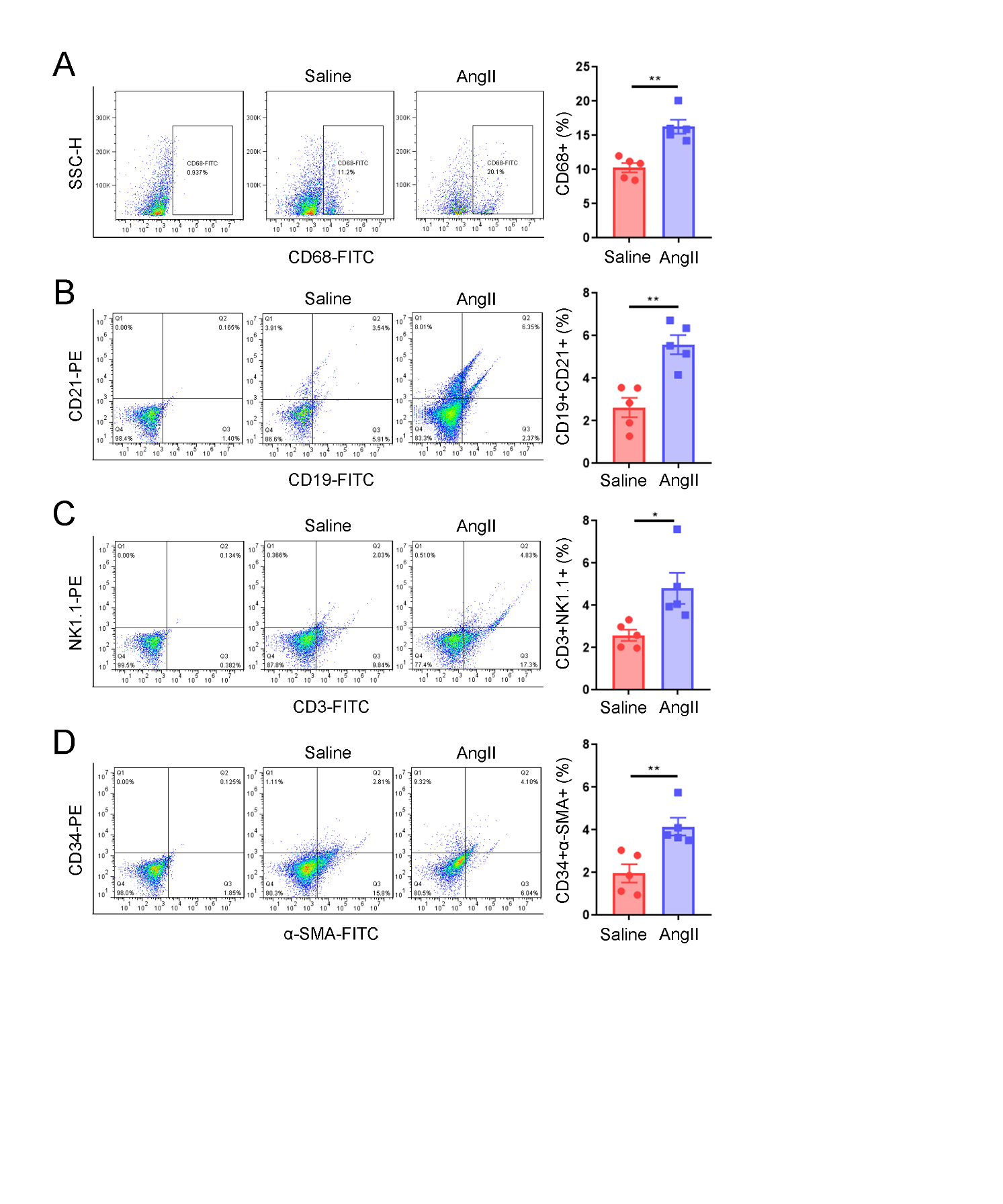


**Supplementary information, Fig. S3. FACS analysis of cell heterogeneities during AAA progression**

**A-D,** FACS analysis of different cell types isolated from control (n=5) and Ang II-infused mice (n=5) labeled with following markers: (**A**) CD68-FITC, (**B**) CD19-FITC and CD21-PE, (**C**) CD3-FITC and NK1.1-PE, and (**D**) CD34-PE and α-SΜΑ-FITC. The quantitative data were shown as mean ± SEM, **p*<0.05, ***p*<0.01, ****p*<0.001, *****p*<0.0001 by t test.


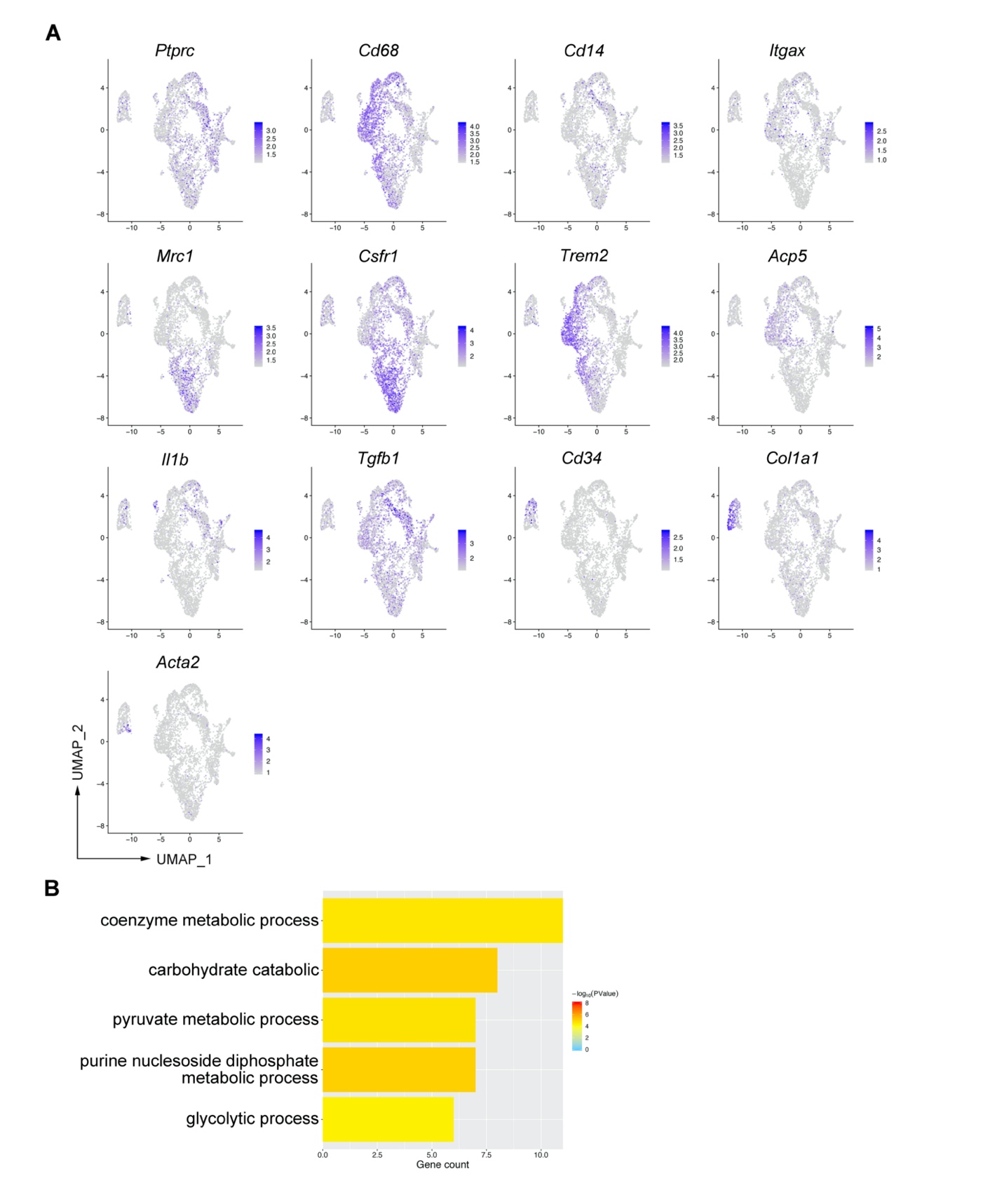


**Supplementary information, Fig. S4. Identification of macrophage subtypes.**

**(A)** The expression of marker genes exhibited on macrophages Uniform manifold approximation and projection (UMAP) plot (gene expression log-normalized by Seurat). **(B)** Gene ontology (GO) pathway enrichment bar plot of DEGs in *Trem2^+^Acp5^+^* macrophages.


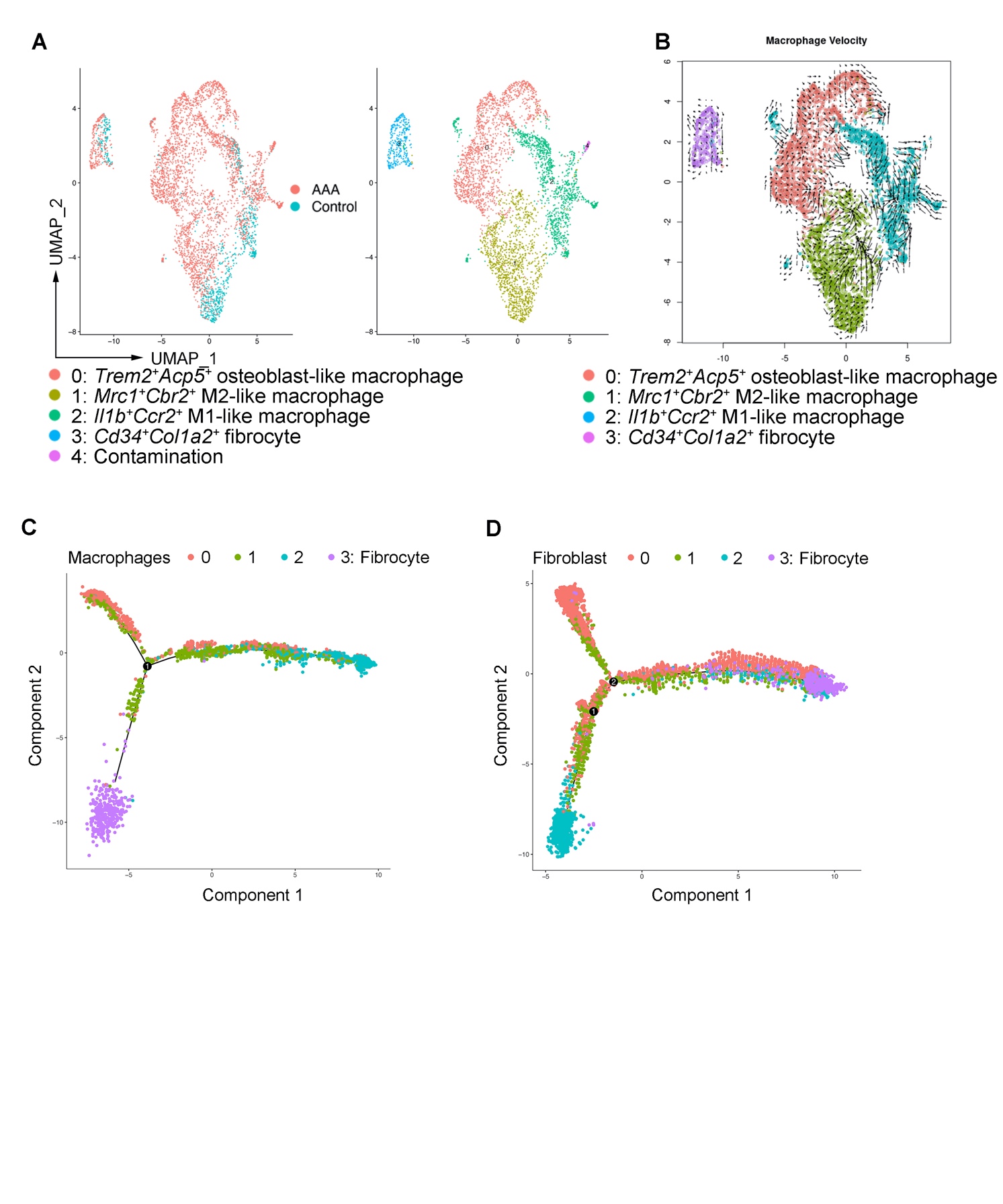


**Supplementary information, Fig. S5. Re-polarization of macrophage subtypes and fibroblasts during AAA pathogenesis.**

**(A)** RNA velocity in macrophage subtypes. **(B)** Pseudo-time plot by trajectory analysis of three clusters of macrophages (Cluster 0: *Trem2^+^Acp5^+^* macrophages; Cluster 1: *Mrc1^+^Cbr2^+^* M2-like macrophages; Cluster 2: *Il1b^+^Ccr2^+^* M1-like macrophages; Cluster 3: *Ptprc*^+^*Col1a2*^+^ fibrocytes). **(C)** Pseudo-time plot by trajectory analysis of three clusters of macrophages (Cluster 0: *Cd34^hi^Acta2^-^* inactivated fibroblast; Cluster 1: *Cd34^low^Acta2^low^* intermediate fibroblast; Cluster 2: *Cd34^hi^Acta2^hi^* myofibroblast; Cluster 3: *Ptprc*^+^*Col1a2*^+^ fibrocytes).


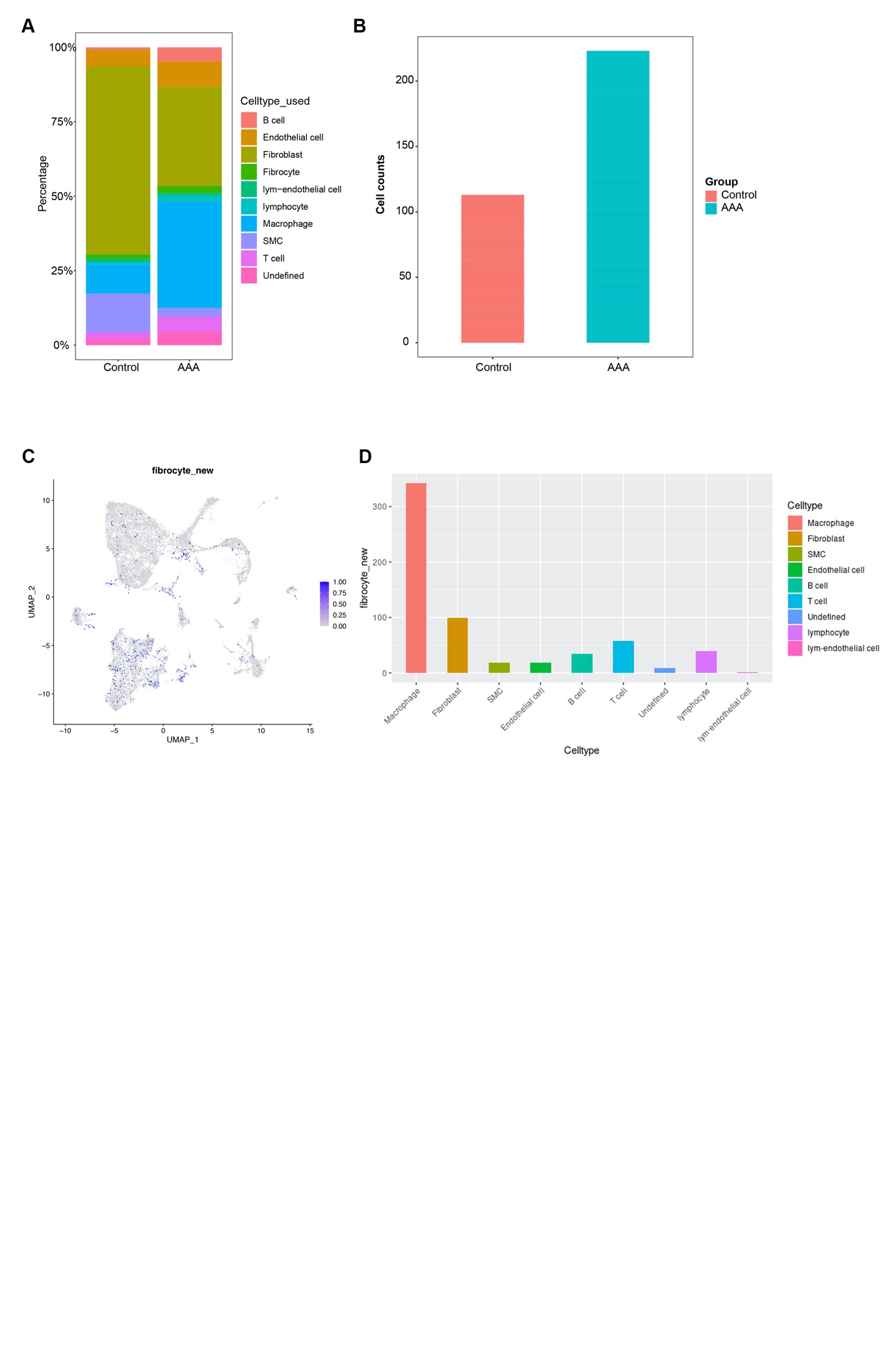


**Supplementary information, Fig. S6. Newly identified fibrocytes by co-expressing *Ptprc* and *Col1a2* in the AAA dataset.**

**(A)** Cell proportions of fibrocytes in AAA mouse and saline control. **(B)** Cell numbers of fibrocytes in AAA mouse and saline control. **(C)** UMAP plot shows the distribution of fibrocytes identified by co-expression of *Ptprc* and *Col1a2.* **(D)** Cell numbers of fibrocytes in each cell type.


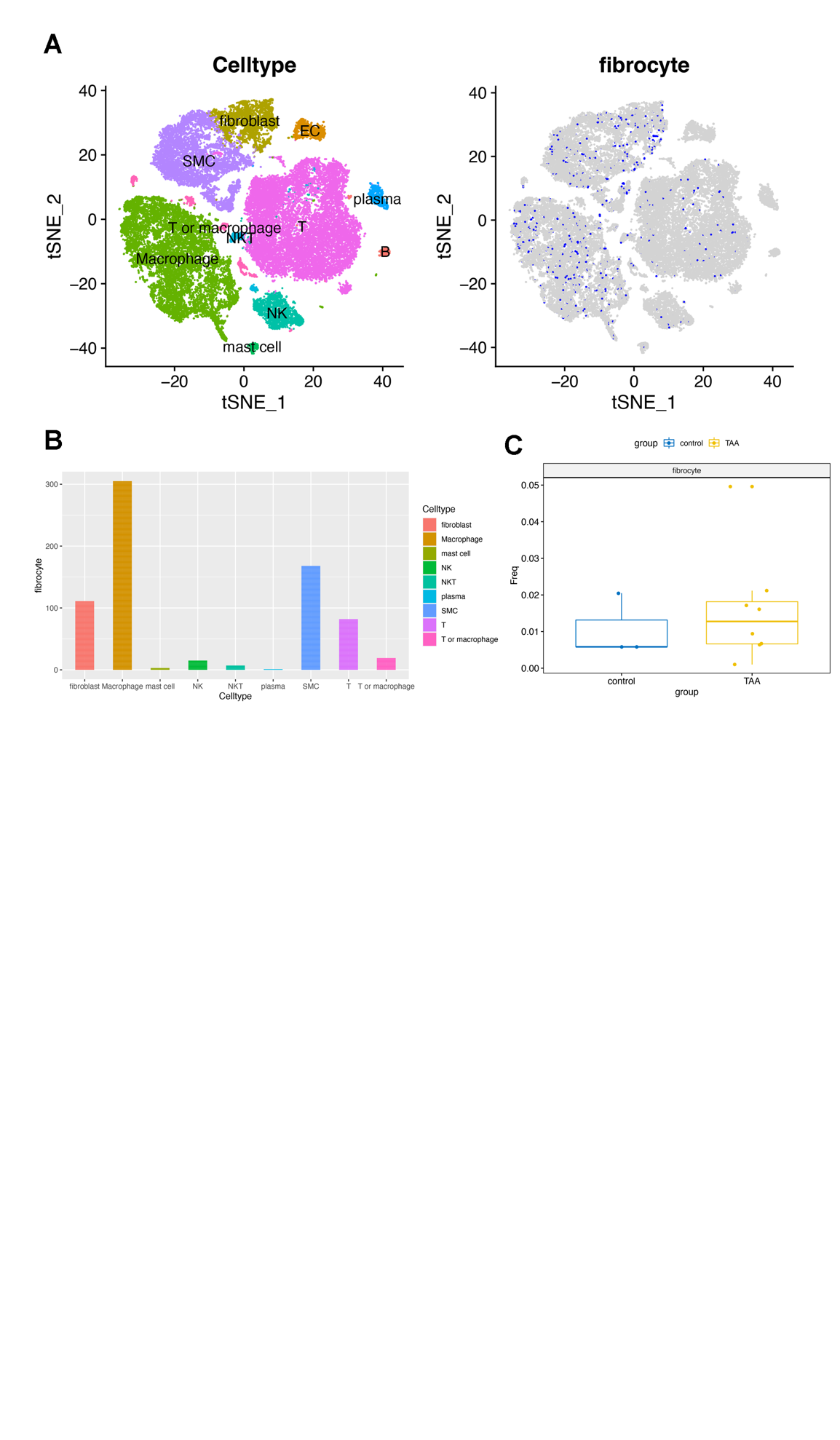


**Supplementary information, Fig. S7. Validation of fibrocytes in human ATAA dataset.**

**(A)** UMAP plot showing aortic cells from ATAA patients and health human with clusters labeled by cell types. The right panel shows the distribution of fibrocytes identified by co-expression of *PTPRC* and *COL1A2.* **(B)** Cell numbers of fibrocyte in each cell type. **(C)** cell proportions of fibrocytes in TAA patients and health controls.


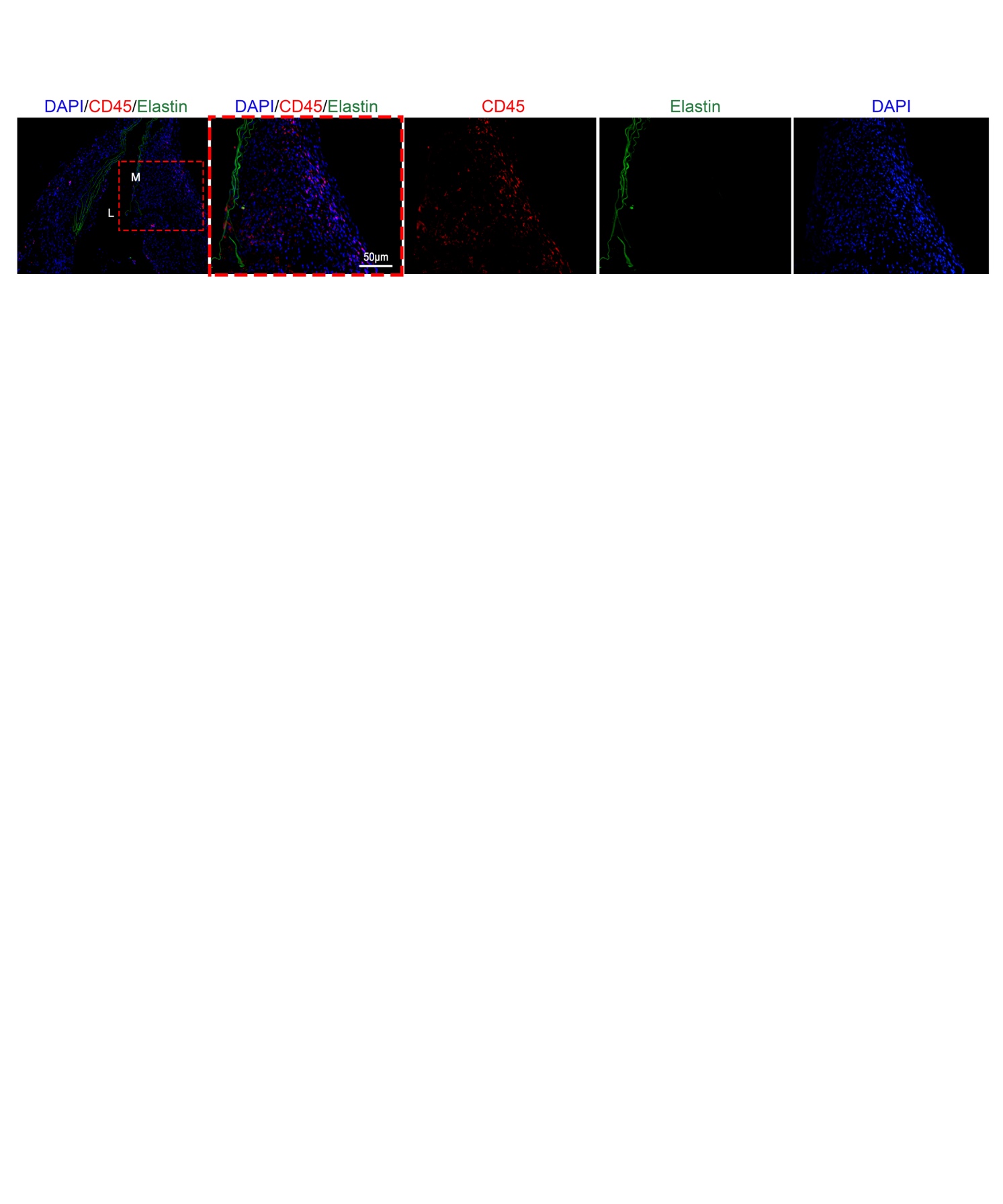


**Supplementary information, Fig. S8. Localization of CD45+ cells in AAA tissues.**

Representative images of immunofluorescence staining of the aneurysm from AngII-induced AAA mice stained with CD45 (red) and elastin (green). L: lumen; M: media.


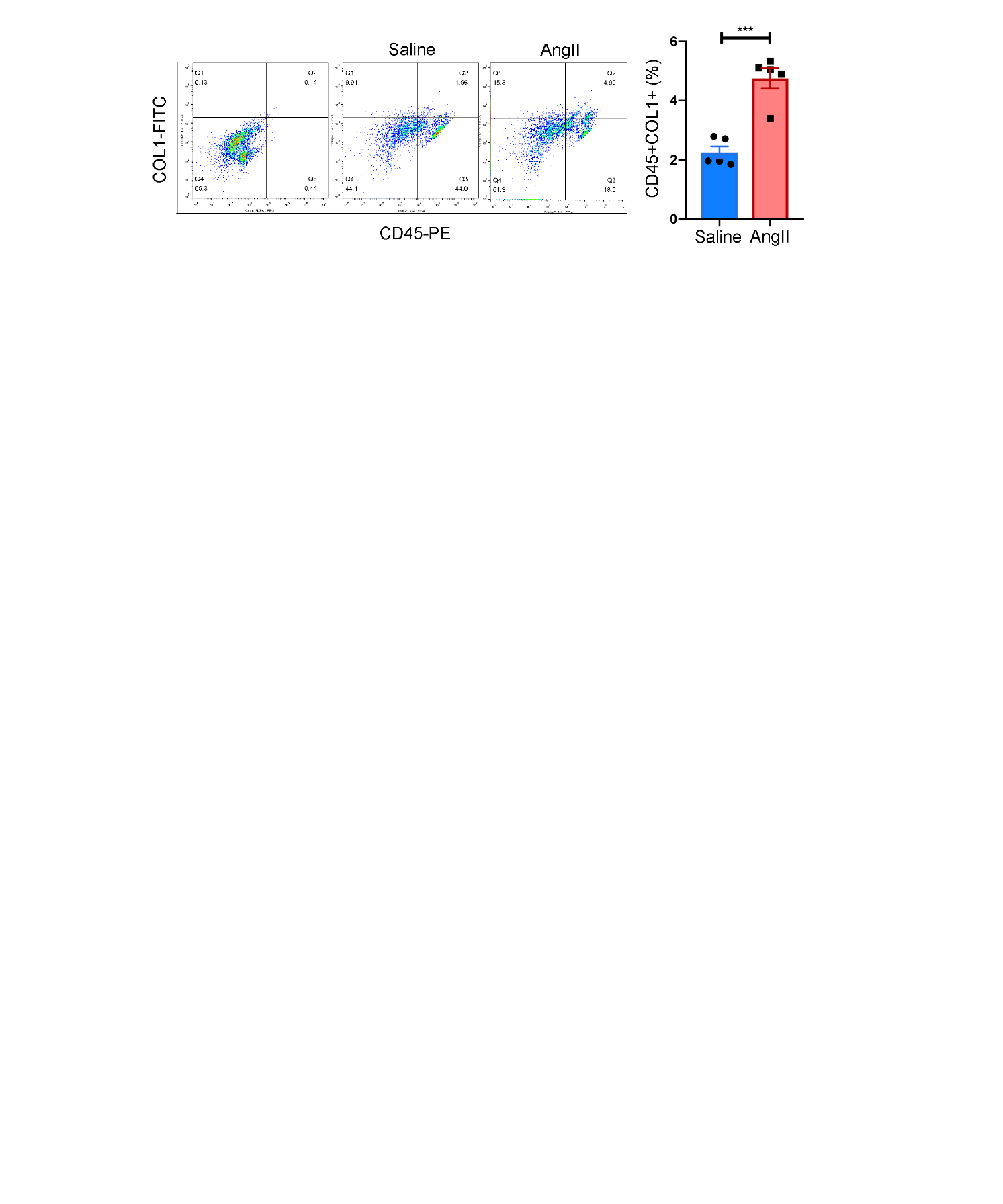


**Supplementary information, Fig. S9. Flow cytometry analysis of fibrocytes during AAA progression**

Flow cytometry analysis of different cell types isolated from saline treated (n=5) and Ang II-infused mice (n=5) labeled with CD45-FITC and COL1-PE. The quantitative data were shown as mean ± SEM, ****p*<0.001 by t test. COL1: Collagen I


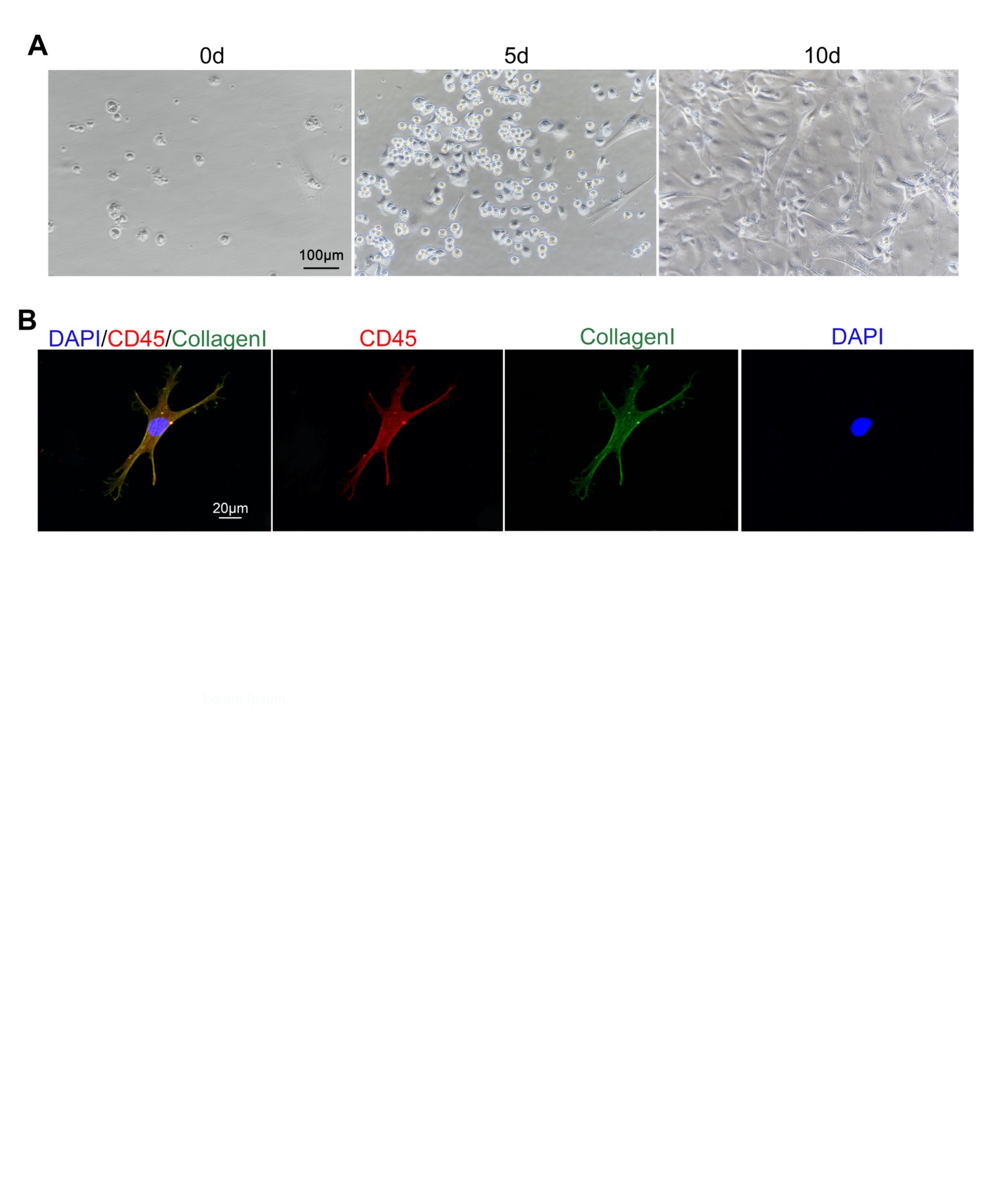


**Supplementary information, Fig. S10. Culture of bone marrow-derived fibrocytes.**

**(A)** Representative images of cultured fibrocytes taken by optical microscopy at day 0, 5, and 10. **(B)** Representative images of immunostaining of cultured fibrocytes with CD45 (red) and collagen I (green) at day 10.


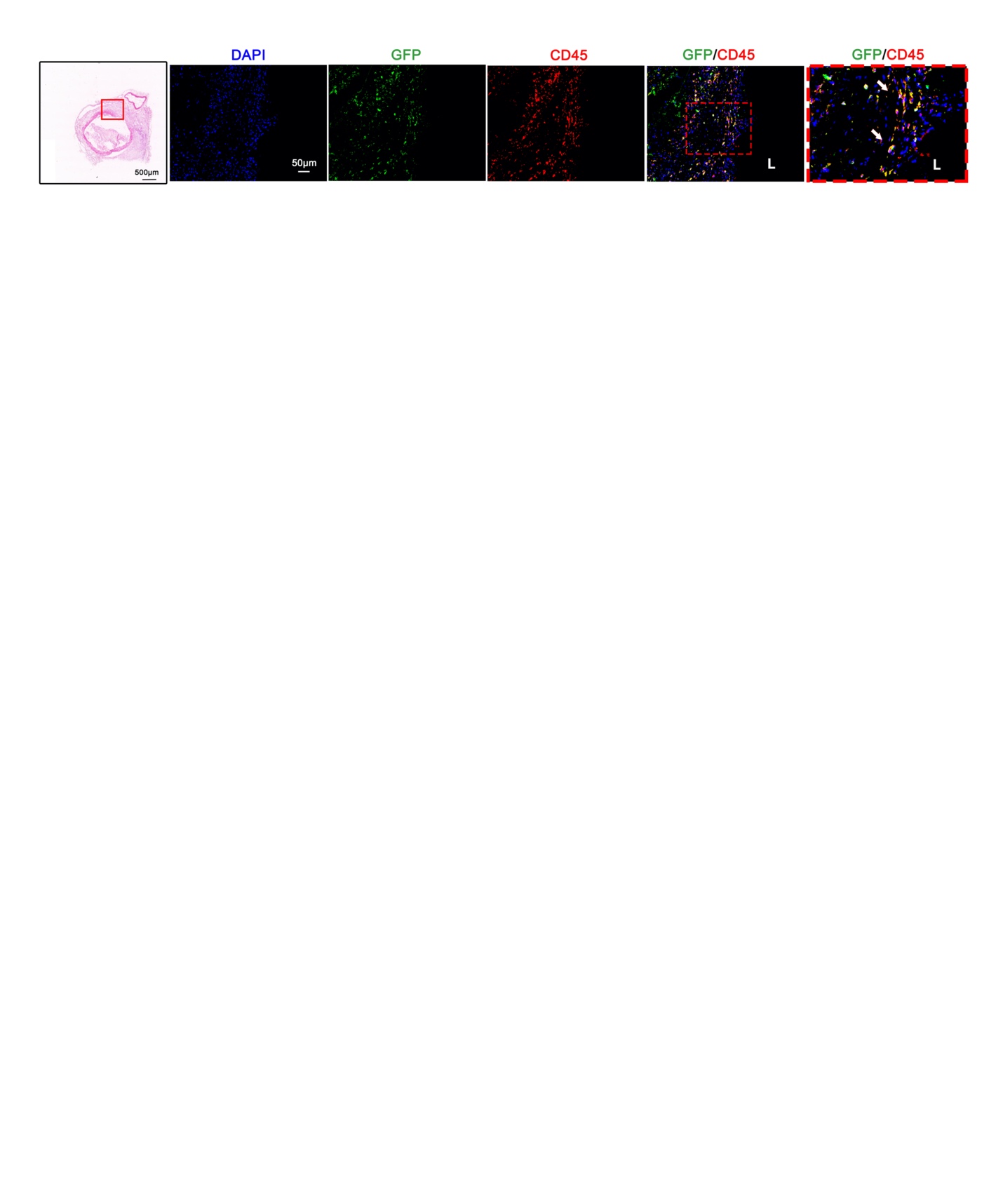


**Supplementary information, Fig. S11. Tracing of GFP-labeled fibrocyte tracing in reconstitution tissue.**

Representative images of H&E and immunofluorescence staining of the aneurysm from AngII-induced AAA mice injected with GFP-labeled fibrocytes. Fibrocytes were stained with CD45 (red) and GFP (green). L: lumen.


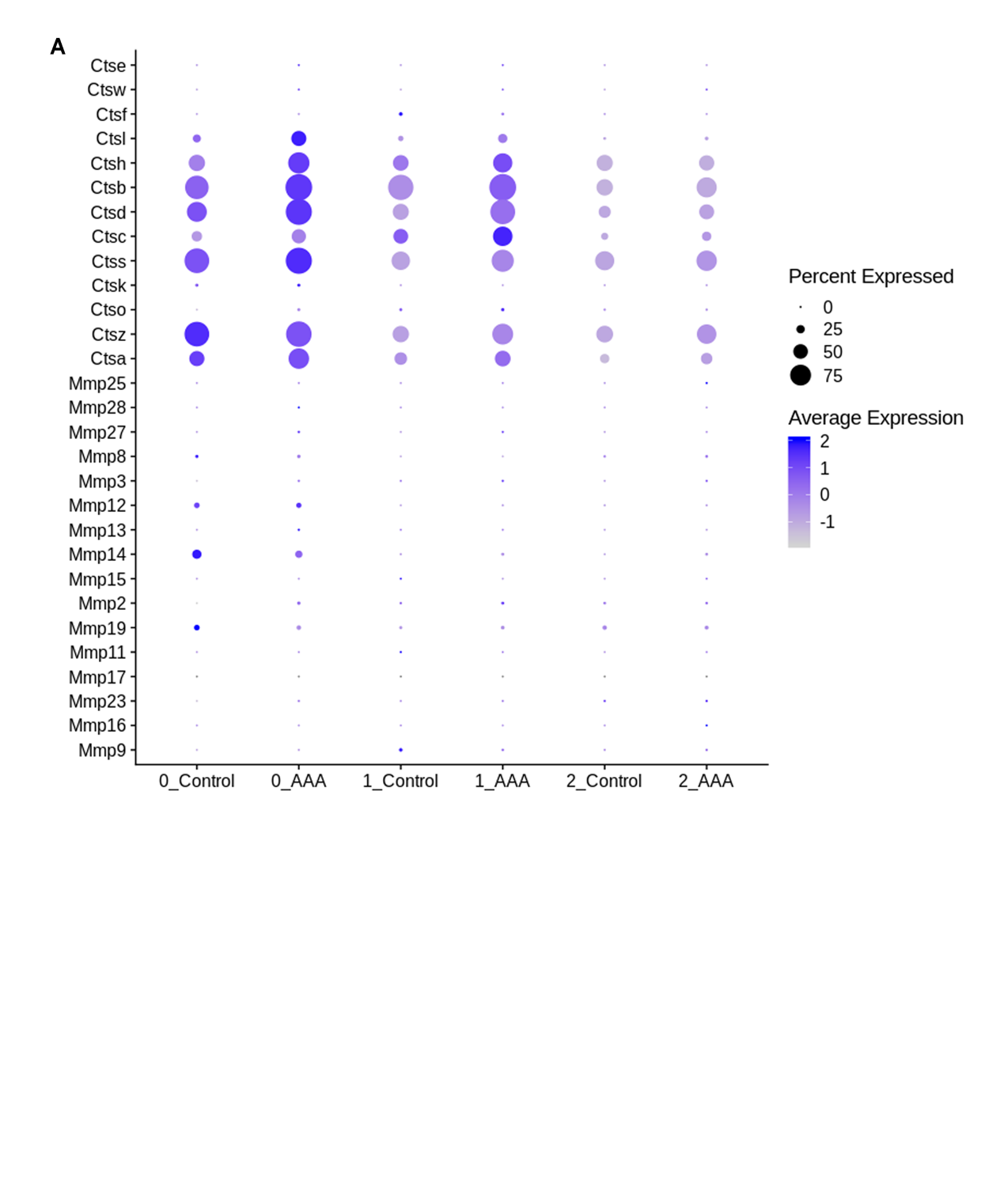


**Supplementary information, Fig. 12. Expression of Cathepsins and matrix metalloproteinases in macrophage subtypes.**

**(A)** Bubble plot of cathepsins and MMPs expression detected by scRNA-seq in three subtypes of macrophages from control and AAA group. (Cluster 0: *Trem2^+^Acp5^+^* macrophages; Cluster 1: *Mrc1^+^Cbr2^+^* M2-like macrophages; Cluster 2: *Il1b^+^Ccr2^+^* M1-like macrophages).
